# Pharmacological profile of dicaffeoylquinic acids and their role in the treatment of respiratory diseases

**DOI:** 10.3389/fphar.2024.1371613

**Published:** 2024-08-22

**Authors:** Matthias Hufnagel, André Rademaekers, Anika Weisert, Hanns Häberlein, Sebastian Franken

**Affiliations:** ^1^ Engelhard Arzneimittel GmbH & Co. KG, Niederdorfelden, Germany; ^2^ Medical Faculty, Institute of Biochemistry and Molecular Biology, University of Bonn, Bonn, Germany

**Keywords:** dicaffeoylquinic acid, isochlorogenic acid, DCQA, pre-clinical evaluation, pharmacological profile, respiratory disease

## Abstract

Dicaffeoylquinic acids (DCQAs) are polyphenolic compounds found in various medicinal plants such as *Echinacea species* and *Hedera helix,* whose multi-constituent extracts are used worldwide to treat respiratory diseases. Besides triterpenes, saponins, alkamides, and other constituents, DCQAs are an important group of substances for the pharmacological activity of plant-derived extracts. Therefore, the pharmacological properties of DCQAs have been studied over the last decades, suggesting antioxidative, anti-inflammatory, antimicrobial, hypoglycaemic, cardiovascular protective, neuroprotective, and hepatoprotective effects. However, the beneficial pharmacological profile of DCQAs has not yet been linked to their use in treating respiratory diseases such as acute or even chronic bronchitis. The aim of this review was to assess the potential of DCQAs for respiratory indications based on published *in vitro* and *in vivo* pharmacological and pre-clinical data, with particular focus on antioxidative, anti-inflammatory, and respiratory-related effects such as antitussive or antispasmodic properties. A respective literature search revealed a large number of publications on the six DCQA isoforms. Based on this search, a focus was placed on 1,3-, 3,4-, 3,5-, and 4,5-DCQA, as the publications focused mainly on these isomers. Based on the available pre-clinical data, DCQAs trigger cellular mechanisms that are important in the treatment of respiratory diseases such as decreasing NF-κB activation, reducing oxidative stress, or activating the Nrf2 pathway. Taken together, these data suggest an essential role for DCQAs within herbal medicines used for the treatment of respiratory diseases and highlights the need for the identifications of DCQAs as lead substances within such extracts.

## 1 Introduction

**FIGURE 1:**
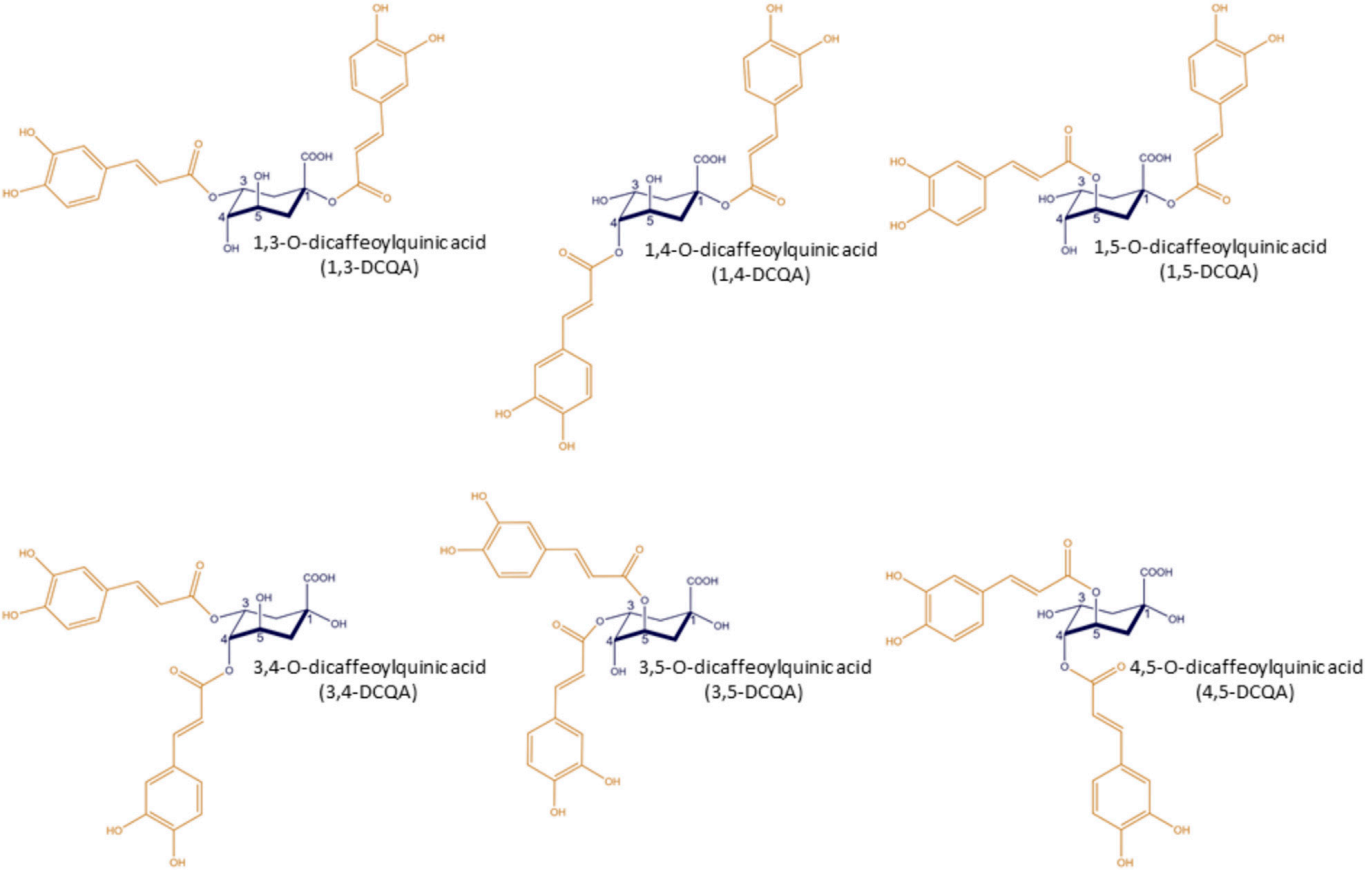
Isoforms of dicaffeoylquinic acids. (Figure created using ChemDraw Pro 8.0, by Perkin Elmer, Waltham, Massachusetts, U.S.).

Dicaffeoylquinic acids (DCQAs) are naturally occurring polyphenols and are esters composed of quinic acid and two caffeoyl acid moieties. Taking into account all possible structures with ester bonds on the quinic acid ring structure, a total of six different DCQAs are known, namely, 1,3-, 1,4-, 1,5-, 3,4-, 3,5- and 4,5-DCQA (Figure 1). The DCQAs shown in Figure 1 and the data on DCQAs used in this review follow the IUPAC nomenclature. This is noteworthy as many publications present structures that do not comply to the IUPAC system or do not specify the structure of the DCQA used. In this review, the authors have aligned the nomenclature within the cited studies to the IUPAC nomenclature. In general, DCQAs are secondary metabolites and are found in various plants, both used for dietary purposes, such as coffee (Behne et al., 2023), and in medical plants, e.g., *Hedera helix* (Bezruk et al., 2020). Furthermore, Wang and colleagues described DCQA being a main constituent in well certain well-known traditional Chinese medicine formulation, e.g., *Shuang-Huang-Lian* and *Reduning* (Wang et al., 2020). Both are suggested to be effective against respiratory infections (Ma et al., 2017; Cao et al., 2020). Biosynthesis of monocaffeoylquinic acid or chlorogenic acid occurs by a combination of the shikimic acid pathway and the phenylpropanoid pathway, as chlorogenic acid represents an intermediate in the biosynthesis of lignin. Currently, the biosynthesis of DCQA is not yet fully understood, however, it is hypothesized to be a result of monocaffeoylquinic acid acylation with caffeoyl-CoA (Behne et al., 2023). Some toxicological data on DCQAs regarding a safe use is available, specifically on acute toxicity (dermal and oral) and neurotoxicity, which did not identify any adverse findings. The data indicates a low potential for immunotoxicity as a pure substance without protein interaction, but this requires further investigation. Additionally, a sub-chronic toxicity study on a combination of DCQAs and chlorogenic acids is available, which did not identify any safety-related concerns. In addition, *in silico* methods for mutagenicity and carcinogenicity yielded no alerts regarding these endpoints. However, there is currently a lack of data on reproductive toxicity and teratogenicity. From a pharmacological perspective, DCQAs confer various effects and are linked with antioxidative, cardiovascular protective, antibacterial, antiviral, hypoglycemic, hepatoprotective, anti-inflammatory, and neuroprotective effects (Wang et al., 2020). To the best of the author’s knowledge, no review has linked the pharmacological data of DCQAs to their potential for treating inflammatory respiratory diseases such as acute or chronic bronchitis. This paper provides an overview of the available pharmacokinetic and pharmacodynamic data on DCQAs, and highlights their potential for treating such respiratory diseases.

## 2 Literature research

To assess all available information from DCQAs, a list of search queries containing two descriptors was generated. The substance’s identity was defined using the first descriptor, which includes the IUPAC term, available synonyms, as well as the CAS and EC number. The second descriptor defined the pharmacological impact and was categorized into pharmacokinetic, anti-inflammatory, antioxidant, immunomodulatory, and effects related to respiratory disease (Table 1). Each descriptor was paired with the keywords of descriptor 2 for searching. The searches were conducted using the PubMed database to obtain our findings.

**TABLE 1:** Summary of search queries applied for data evaluation.

Descriptor 1	Descriptor 2
“1,3-Dicaffeoylquinic acid” OR Cynarin OR ″30,964-13-7″ OR ″845-639-3″	*Pharmacokinetic*: (metabolism OR ADME OR pharmacokinetic)
“1,4-Dicaffeoylquinic acid” OR ″1,182-34-9″ OR ″214-655-7″	*Anti-inflammatory effect*: (“inflammation” OR “anti-inflammatory” OR “antiinflammatory")
“1,5-Dicaffeoylquinic acid” OR ″19,870-46-3″ OR ″815-081-5″	*Anti-oxidative effect*: (“antioxidative” OR “anti-oxidative” OR “oxidative stress")
“3,4-Dicaffeoylquinic acid” OR “isochlorogenic acid C″ OR ″57,378-72-0″	*Immunomodulation*: (“immunomodulation” OR “immunomodulative” OR “immune system")
“3,5-Dicaffeoylquinic acid” OR “isochlorogenic acid A″ OR ″2,450-53-5″ OR ″815-082-0″	*Respiratory disease-related*: (“bronchodilatation” OR “secretolytic” OR “anti-tussive” OR “antitussive")
“4,5-Dicaffeoylquinic acid” OR “isochlorogenic acid B″ OR ″14,534-61-3″	

Literature research was performed applying the search queries in PubMed. Descriptor 1 defines the substance identity applying the IUPAC term, available synonyms, as well as the CAS and EC number. Descriptor 2 defines the pharmacological impact and was categorized into pharmacokinetic, anti-inflammatory, antioxidant, immunomodulatory, and effects related to respiratory disease.

**FIGURE 2:**
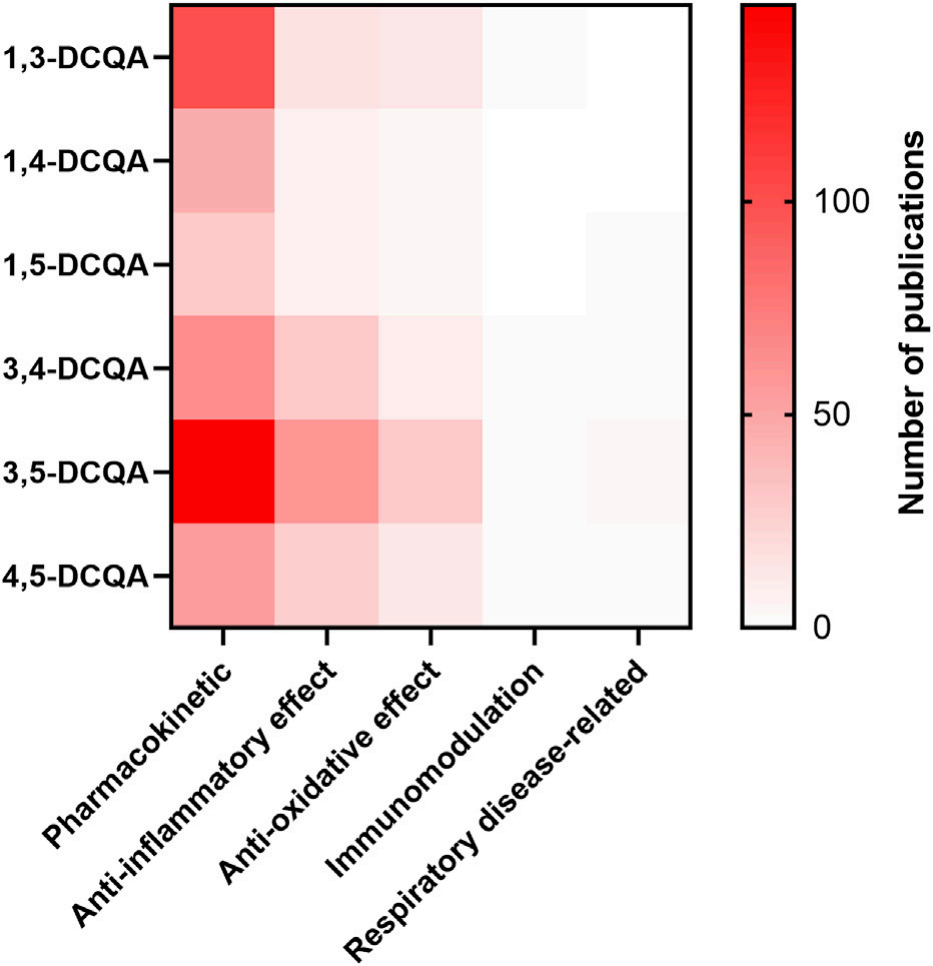
Heatmap of the results on the search queries used. The number of publications is shown as a function of the substance and the descriptor used. The applied descriptors are listed in Table 1.

Using the criteria outlined in Table 1, we obtained a total of 673 search results, without disregarding the possibility of duplicate hits across queries. The distribution of results by criterion is further illustrated in Figure 2. According to Figure 2, pharmacological data is predominantly accessible on pharmacokinetics, followed by inflammation and oxidative stress/antioxidant capacity. Additionally, there is a lack of information on 1,4- and 1,5-DCQA. Therefore, this review will focus on 1,3-, 3,4-, 3,5- and 4,5-DCQA, and their pharmacokinetic, anti-inflammatory and antioxidant properties. Due to the scope of this review, the impact of DCQAs on respiratory disease-related endpoints was also evaluated, despite the limited availability of data. To compare the pharmacodynamics of isomers and provide an effect-inducing range of DCQAs, we utilized point of departures (PoDs) from relevant studies. These PoDs were either IC/EC_50_ values, if accessible, or the concentration or dose causing a statistically significant effect on an endpoint. All *in vitro* data are expressed as concentration (µM), whereas *in vivo* data are reported as dose (mg/kg bw/d).

## 3 Pharmacokinetic

Data on the pharmacokinetic of DCQAs are published diversified including both, *in vitro* and *in vivo* studies. However, data on single DCQAs is scarce and mainly extracts containing DCQAs were investigated. Therefore, within this review it was focused on data relevant for the absorption, distribution, metabolism, and elimination of DCQAs mainly applied as constituent within an extract. Data of *in vivo* pharmacokinetic studies were only considered for oral administration of a test item and are summarized in Table 2. Overall, no relevant differences between the different DCQA isoforms were apparent regarding pharmacokinetic endpoints.

**TABLE 2:** Summary of available *in vivo* pharmacokinetic data from studies with oral administration of DCQA-containing extracts in rats.

Substance	Extract	Dose	Dose DCQA^a^ [mg/animal]	c_max_ [µg/mL]	t_max_ [min]	AUC_0-∞_[µg^a^ h/mL]	t_1/2_ [h]	V_z_ [L/kg]	CL [L//kg]	f^a^ (%)	Reference
3,4-DCQA	Mix of *Flos Lonicerae (Lonicera japonica* Thunb.*)* and *Fructus Forsythiae (Forsythia Suspense)*	25 g/kg bw	24.72	0.06674	10	12.802	9.1	n/a	n/a	0.8	Zhou et al. (2015b)
	*Flos Lonicerae (Lonicera japonica* Thunb.*)*	10 mL/kg bw	94.5	0.074	53	51.145	10.9	n/a	n/a	0.8	Zhou et al. (2013)
	*Flos Lonicerae (Lonicera japonica* Thunb.*)*	10 mL/kg bw	75.75	0.0735	65	88.148	11.2	n/a	n/a	1.7	Zhou et al. (2013)
	*Flos Lonicerae (Lonicera japonica* Thunb.*)*	10 mL/kg bw	72.25	0.0511	55	37.048	12.7	n/a	n/a	0.8	Zhou et al. (2013)
	*Flos Lonicerae (Lonicera japonica* Thunb.*)*	10 mL/kg bw	75.5	0.056	35	64.954	6.9	n/a	n/a	1.3	Zhou et al. (2013)
	*Flos Lonicerae (Lonicera japonica* Thunb.*)*	10 mL/kg bw	58.5	0.0555	30	37.807	10.1	n/a	n/a	1.0	Zhou et al. (2013)
	*Flos Lonicerae (Lonicera japonica* Thunb.*)*	10 mL/kg bw	63.25	0.0317	27.5	29.113	12.5	n/a	n/a	0.7	Zhou et al. (2013)
	Mix of *Flos Lonicerae (Lonicera japonica* Thunb.*)* and *Fructus Forsythiae (Forsythia Suspense)*	10 mL/kg bw	6.75	0.03902	10	8.53482	9.4	n/a	n/a	1.9	Zhou et al. (2014a)
	*Ainsliaea fragrans* Champ	0.16 g/kg bw	3.504	0.062	30	0.166,333	1.96	347	118	0.1	Su et al. (2014)
	*Flos Lonicerae (Lonicera japonica* Thunb.*)*	10 mL/kg bw	6.47	0.00479	15	1.3766	4.9	n/a	n/a	0.3	Zhou et al. (2014b)
	Mix of *Flos Lonicerae (Lonicera japonica* Thunb.*)* and *Fructus Forsythiae (Forsythia Suspense)*	10 mL/kg bw	6.47	0.0125	12	2.3276	6.8	n/a	n/a	0.5	Zhou et al. (2014b)
3,5-DCQA	*---*	18 mg/kg bw	3.6	7.05	22.01	82.58	0.5	9.52	13.2	22.6^b^	Cen et al. (2017)
	Mix of *Flos Lonicerae (Lonicera japonica* Thunb.*)* and *Fructus Forsythiae (Forsythia Suspense)*	25 g/kg bw	13.025	0.0705	10	3.25	5.9	n/a	n/a	0.4	Zhou et al. (2015b)
	*Flos Lonicerae (Lonicera japonica* Thunb.*)*	10 mL/kg bw	47.25	0.081	31.7	51.271	6.3	n/a	n/a	1.6	Zhou et al. (2013)
	*Flos Lonicerae (Lonicera japonica* Thunb.*)*	10 mL/kg bw	38.25	0.0701	60	52.813	8.4	n/a	n/a	2.1	Zhou et al. (2013)
	*Flos Lonicerae (Lonicera japonica* Thunb.*)*	10 mL/kg bw	34.75	0.0572	40	31.219	10.1	n/a	n/a	1.3	Zhou et al. (2013)
	*Flos Lonicerae (Lonicera japonica* Thunb.*)*	10 mL/kg bw	37.75	0.0816	25	50.509	7.1	n/a	n/a	2.0	Zhou et al. (2013)
	*Flos Lonicerae (Lonicera japonica* Thunb.*)*	10 mL/kg bw	32.75	0.121	30	44.537	9.2	n/a	n/a	2.0	Zhou et al. (2013)
	*Flos Lonicerae (Lonicera japonica* Thunb.*)*	10 mL/kg bw	33.5	0.0684	30	36.091	12.5	n/a	n/a	1.6	Zhou et al. (2013)
	*Erigeron breviscapus*	5 g/kg bw	9.005	1.033	70.2	7.59	17.6	110	0.0047	1.3	Tian et al. (2017)
	*Dipsacus*. *asper* Wall. ex C.B. Clarke (raw)	75.6 g/kg bw	0.369	0.20706	30	0.33623	2.94	n/a	n/a	1.4	Tao et al. (2016)
	*Dipsacus asper* Wall. ex C.B. Clarke (processed)	75.6 g/kg bw	0.254	0.28065	55	0.97698	1.81	n/a	n/a	5.8	Tao et al. (2016)
	Mix of *Flos Lonicerae (Lonicera japonica* Thunb.*)* and *Fructus Forsythiae (Forsythia Suspense)*	10 mL/kg bw	4.325	0.05277	11.67	2.70876	5.1	n/a	n/a	0.9	Zhou et al. (2014a)
	*Ainsliaea fragrans* Champ	0.16 g/kg bw	3.162	0.0535	30	0.10049	1.49	429	180	0.05	Su et al. (2014)
4,5-DCQA	*Ainsliaea fragrans* Champ	0.16 g/kg bw	1.748	0.0241	16.8	0.01907	1.94	336	543	0.02	Su et al. (2014)
	*Erigeron breviscapus*	5 g/kg bw	2.98	0.08752	6.6	3.065	27.7	4.3	0.0002	1.5	Tian et al. (2017)
	*Flos Chrysanthemi*	10 g/kg	12.95	0.10123	24	0.40453	0.24	n/a	n/a	0.05	Jia et al. (2020)

^a^ according to Equation 1.

^b^ not calculated via estimation but given within study.

With exception of Su et al. (2014) which do not specify the used rat strains, all studies were performed in Sprague-Dawley rats. c_max_: maximum concentration, t_max_: time to reach maximum concentration, AUC_0-∞_: area under the concentration-time curve from zero to infinity, t_1/2_: elimination half-time, V_Z_: volume of distribution during terminal phase, CL: clearance, f: bioavailability, n/a: not available.

### 3.1 Absorption

#### 3.1.1 In vitro-based data on DCQA absorption

Compound stability during digestion is a critical factor for substance absorption. Takenaka et al. (2000) investigated the stability of 3,5-DCQA after artificial digestion of 2 h at 37 °C in artificial gastric juice followed by incubation in artificial intestinal fluid at the same temperature for 2 h. It was observed that 3,5-DCQA exhibited a stability of almost 100% subsequent to artificial digestion (Takenaka et al., 2000). Another study investigated the impact of artificial digestion on DCQAs within yarrow extracts, both native and enriched (Villalva et al., 2022). Under oral conditions, no degradation of the investigated DCQAs was observed. However, gastric conditions caused a loss of roughly 20% for 3,5- and 4,5-DCQA. Subsequent intestinal conditions resulted in 63%–67% degradation of 3,5-DCQA. During this digestion step, the amount of 3,4- and 4,5-DCQA increased significantly (Villalva et al., 2022). The authors suggest that the observed phenomenon may be due to isomerization resulting from a pH shift between gastric and intestinal conditions. After undergoing artificial digestion, the total DCQA content of the extract was still 90% compared to the undigested extract (Villalva et al., 2022). D'Antuono and colleagues (2015) also reported the isomerization effect of 3,5-DCQA during digestion, which was further enhanced in the presence of 3,4- and 4,5-DCQA (D’Antuono et al., 2015). Absorption of 3,4- and 3,5-DCQA was investigated *in vitro* using Caco-2 cell models demonstrating a time-dependent absorption as well as P_app_ values of 1–2.5 × 10^−6^ cm s^-1^, indicating a moderate absorption (Zhou et al., 2015a; Villalva et al., 2022). Within two studies, DCQAs were not detected in the basolateral fraction of Caco-2 cells after 6 h. However, due to the presence of caffeic and coumaric acid cellular metabolic activity within the applied assay was thought to be the cause for this observation (D’Antuono et al., 2015; Villalva et al., 2022). Mechanistically, Zhou and colleagues (2015a) suggested passive diffusion as driving force behind DCQA absorption (Zhou et al., 2015a).

#### 3.1.2 In vivo-based data in DCQA absorption

**FIGURE 3:**
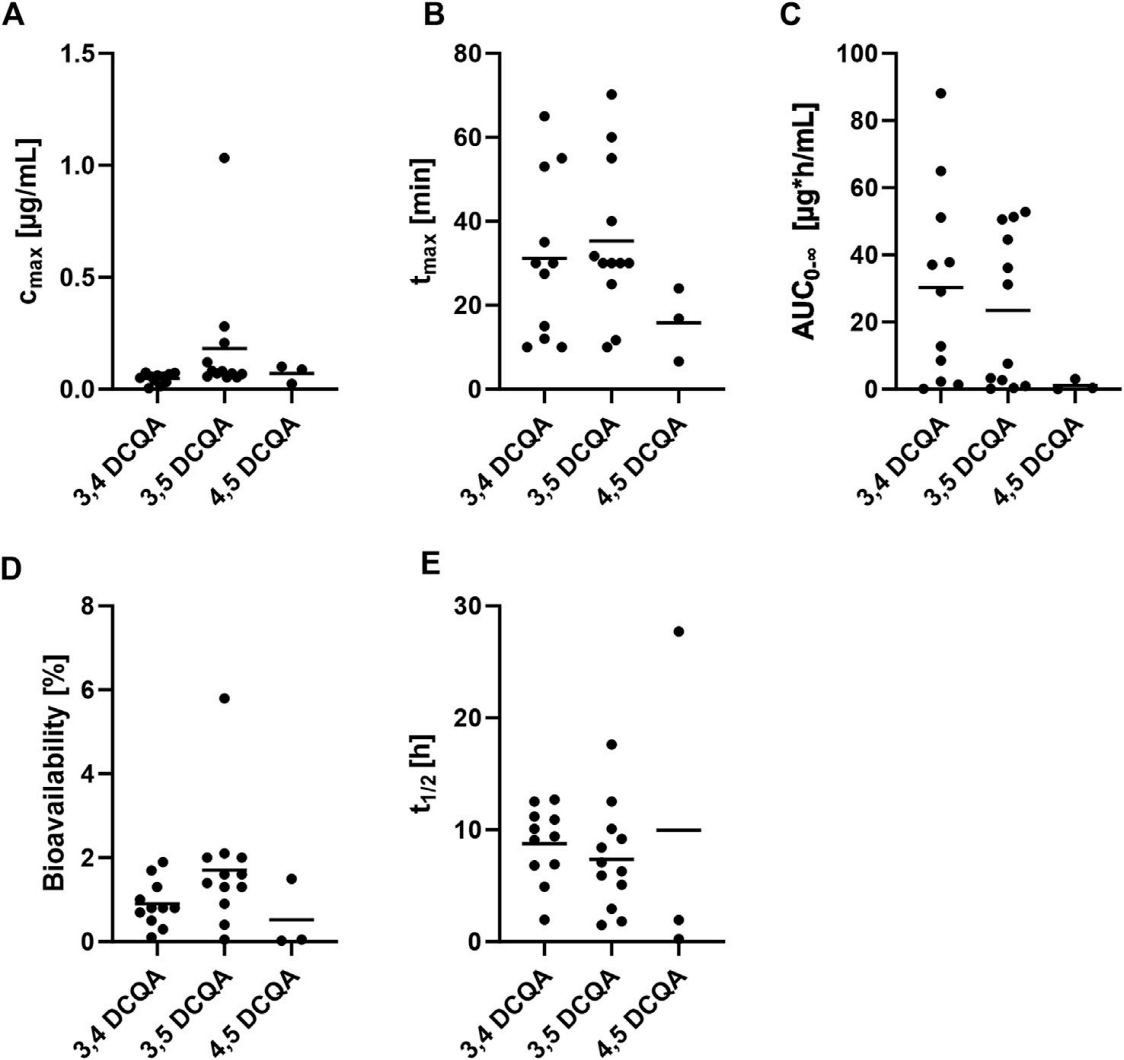
Review of available pharmacokinetic data on DCQA containing extracts applied via oral administration to rats. Data is displayed in a scatter plot where each dot represents a data point from a single study. The lines within the plot indicate the mean of the respective data. **(A)** maximum plasma concentration (c_max_), **(B)** time to reach maximum concentration (t_max_), **(C)** area under the concentration-time curve from zero to infinity (AUC_0-∞_), **(D)** bioavailability, **(E)** elimination half-time (t_1/2_). Table 2 summarizes the references to the studies used for this illustration.

Several *in vivo* pharmacokinetic studies were conducted on rats that were administered mainly DCQA-containing extracts, with only one study using 3,5-DCQA as a single substance (Table 2). A bioavailability (f) of 22.6%, maximum concentration (c_max_) of 7.08 μg/mL, time to reach maximum concentration (t_max_) of 22.01 min, and area under the concentration-time curve from zero to infinity (AUC_0-∞_) of 82.58 µg*h/mL were observed after oral administration of 18 mg/kg bw 3,5-DCQA to rats (Cen et al., 2017). However, these observations were not reproduced when applying extracts containing 3,5-DCQA with observing higher absorption after applying the single substance (Figure 3). A total of twelve pharmacokinetic studies were conducted, administering extracts containing 3,5-DCQA orally to rats. The results showed that c_max_ was in a range of 0.05–1.0 μg/mL, t_max_ ranged from 10 to– 70.2 min, and AUC_0-∞_ was in a range of 0.1–52.8 µg*h/mL. However, it was not possible to properly calculate the bioavailability (f) according to Cen et al. (2017), which is defined as (AUC_po_ * dose_iv_)/(AUC_iv_ * dose_po_), using the available data on DCQA-containing extracts. Therefore, it was assessed as an assumption (Equation 1). The applied DCQA dose was calculated based on the given dosage and the bodyweight of the rats mentioned in the respective studies. If the bodyweight was not provided, a rough estimation of 200 g was used. V_blood_ refers to the total blood volume in rats, which was estimated to be approximately 15 mL in this assumption.

$$f=\frac{{AUC}_{0-\infty }*V_{blood}}{\text{Dose}\;\left(\text{DCQA}\right)}$$ (1)

Based on the calculation provided, the bioavailability of 3,5-DCQA was estimated to be f = 1.7 ± 1.4% (range: 0.05%–5.77%) after oral administration of the respective extracts. When comparing c_max_, t_max_, AUC_0-∞_, and f among the DCQA isoforms (Figures 3A–D), similar results were observed for 3,4-, 3,5-, and 4,5-DCQA in terms of c_max_, t_max_, and f. However, AUC_0-∞_ of 4,5-DCQA was lower when compared to 3,4- and 3,5-DCQA. It is worth noting that there was a limited amount of data available for 4,5-DCQA (n = 3), while data for 3,4-DCQA (n = 10) and 3,5-DCQA (n = 12) were more frequent. Having reviewed all the relevant data on absorption, the authors suggest that the uptake of DCQAs is not targeted towards any particular isoform and occurs swiftly and efficient enough to result in pharmacological benefits.

### 3.2 Distribution

The distribution of 3,5-DCQA and its metabolites was assessed by Gong et al. (2020) after oral administration of 200 mg/kg bw to male Sprague-Dawley rats (Gong et al., 2020). Two di-methylated DCQAs were found in the spleen, whereas only the 3,5-DCQA was found in the heart. Moreover, the parent compound and ten metabolites were detected within the kidney. 3,5-DCQA, along with two di-methylated metabolites and the glucuronic acid conjugate of 3,5-DCQA, were found in the lungs and liver. The metabolites that were observed to be most distributed across the examined tissue were the aforementioned 3,5-DCQA and two di-methylated metabolites, leading the authors to suggest that these compounds might exert pharmacological effects (Gong et al., 2020). In another study, female rats were given *A. fragrans* extract of the aerial parts orally, which contained 1,3-, 1,5-, 3,4-, 3,5-, and 4,5-DCQA (Su et al., 2014). Within 1 h, DCQAs reached their peak value and were predominantly present in the reproductive organs, liver, lungs, and kidneys (Su et al., 2014). Both studies highlight that DCQAs are distributed to the target tissue of this review, namely, the lung.

### 3.3 Metabolism

Generally, the metabolic fate of DCQAs suggests two pathways (Wang et al., 2017; Gong et al., 2020; Zhao et al., 2022). The first is degradation into quinic and caffeic acid since the ester bond appears to be easily hydrolyzed. Subsequently, phase I and II metabolism follows. The second pathway suggests a direct phase I and II metabolism of DCQAs themselves. The occurrence of both pathways has been observed within metabolic profiling of 3,5-DCQA in rat plasma, feces, and urine after single oral or intravenous administration of 50 mg/kg bw to male Sprague-Dawley rats (Wang et al., 2017). This mechanism has been reproduced by analyzing rat plasma after oral administration of 200 mg/kg bw 3,5-DCQA to male Sprague-Dawley rats (Gong et al., 2020). The most apparent metabolic pathways included hydrolysis, methylation, hydrogenation, dehydroxylation, glucuronidation, as well as glycine, cysteine, and sulfate conjugation (Wang et al., 2017; Gong et al., 2020). A metabolic profiling study in male Sprague-Dawley rats administered 200 mg/kg bw of 3,4-DCQA demonstrated the existence of the same pathways, except for the cysteine conjugation, resulting in a total of 67 identified metabolites (Zhao et al., 2022). Wang and colleagues (2017) furthermore reported that the ester bond could hydrolyze easily, the α,β-unsaturated carbonyl group is susceptible to reduction, and hydroxyls within the catechol group are likely to undergo methylation. Additionally, the study showed that phase II conjugates can be formed by intermediate via conjugation of glutathione, cysteine, glycine, sulfate or glucuronic acid (Wang et al., 2017).

### 3.4 Elimination

Since metabolites were found in urine and faeces, elimination via both ways is possible (Wang et al., 2017; Gong et al., 2020; Zhao et al., 2022). Within *in vivo* pharmacokinetic studies applying DCQA containing extracts to rats, the elimination half-time (t_1/2_) was assessed (Figure 3E). Taken all available data together, similar t_1/2_ were obtained for the three isoforms 3,4-, 3,5-, and 4,5-DCQA ranging from 7.4 to 10 h, indicating moderate elimination time. Clearance was explored in several pharmacokinetic studies, but no patterns could be discerned within this review because the respective values differed significantly from one another (Table 2).

## 4 Pharmacodynamic

As mentioned beforehand, DCQAs obtain several published pharmacodynamic relevant properties linked with antioxidative, cardiovascular protective, antibacterial, antiviral, hypoglycemic, hepatoprotective, anti-inflammatory, and neuroprotective effects (Wang et al., 2020). So far, the pharmacodynamic effects of DCQAs have not been evaluated in the context of inflammation-related respiratory diseases such as acute or even chronic bronchitis. Therefore, this chapter aims to highlight the antioxidative, anti-inflammatory, and immunomodulating properties of DCQA. Furthermore, the impact of DCQAs on endpoints directly related to symptoms of respiratory diseases, such as reduction of cough events or enhancement of mucociliary clearance, is evaluated.

### 4.1 Antioxidative properties

Due to the polyphenolic structure of DCQAs, an antioxidative potential is very likely and has been investigated in various studies, including cell-free, cell-based, and *in vivo* studies (Table 3 at the end of the chapter). Specifically, scavenging of free radicals such as reactive oxygen species (ROS) and enhancement of cellular oxidative stress response are involved and play a crucial role in the therapeutic potential of DCQAs.

**TABLE 3:** Summary of studies investigating antioxidative properties of dicaffeoylquinic acids (DCQA) *in vitro* or *in vivo* listing relevant endpoints and point of departures (PoD), which indicate a significant effect on the respective endpoint.

Reference	Model	Trigger	Applied Dose/Concentration	DCQA isoform	Endpoint	PoD
Bouratoua et al. (2018)	Acellular	---	n/a	3,5-DCQA	DPPH	12.87 μg/mL = 24.9 µM (IC_50_)
Cao et al. (2010)	Primary rat astrocytes	Oxygen and glucose deprivation/reperfusion	50 µM	1,5-DCQA	Nrf2 translocation	50 µM
Cao et al. (2017)	Vero cells	EV71 inoculation	100 µM	3,5-DCQA	GR/GPX/G6PD expression	100 µM
Gebhardt and Fausel (1997)	Primary hepatocytes	TBHP	n/a	1,3-DCQA	MDA	23.6 µM (EC_50_)
Iwai et al. (2004)	Acellular	---	n/a	4,5-DCQA	DPPH	5.6 µM (IC_50_)
				3,5-DCQA		6 µM (IC_50_)
				3,4-DCQA		5.8 µM (IC_50_)
Kang et al. (2011)	Hep-G2 cells	TBHP	10–40 µM	3,5-DCQA	DCF	20 µM
					GSH	5 µM
Kim et al. (2005)	SH-SY5Y cells	H_2_O_2_	5–50 µM	3,5-DCQA	GSH	25 µM
Kim et al. (2012)	Rat cortical neurons	Glutamate	0.1–5 µM	3,4-DCQA	DCF	0.1 µM
				3,5-DCQA		
Kim et al. (2022a)	Acellular	---	n/a	3,5-DCQA	DPPH	10.9 µM (EC_50_)
				4,5-DCQA		13.8 µM (EC_50_)
Könczöl et al. (2012)	Acellular	---	0.5–300 μg/mL	3,5-DCQA	DPPH	8.74 μg/mL = 17 µM (IC_50_)
Liang and Kitts (2018)	Caco-2 cells	PMA + INFy	0.2–2 mM	3,4-DCQA	DCF, GSH, Nrf2 translocation	
				3,5-DCQA		200 µM
				4,5-DCQA		
Ma et al. (2015)	Acellular	---	n/a	3,5-DCQA	DPPH	3.63 μg/mL = 7 µM (IC_50_)
				4,5-DCQA		4.01 μg/mL = 7.7 µM (IC_50_)
				3,4-DCQA		4.78 μg/mL = 9.3 µM (IC_50_)
Pagano et al. (2016)	Hep-G2 cells	Free radical initiator	1–12 µM	1,3-DCQA	DCF	2.2 µM (EC_50_)
Park et al. (2009)	Acellular	---	n/a	4,5-DCQA	DPPH	10.09 µM
				3,5-DCQA		9.95 µM
Pérez-García et al. (2000)	Human neutrophils	H_2_O_2_	mM range	1,3-DCQA	DCF	5.2 μg/mL = 10 µM
Psotová et al. (2003)	Acellular	---	0.01–0.1 mM	1,3-DCQA	DPPH	5 µM
	Rat liver homogenate	TBHP	0.1–25 mM		MDA	1.96 mM (IC_50_)
Raineri et al. (2021)	3T3-L1 cells	Hormonal cocktail (MDI)	10 µM	3,5-DCQA	HO-1/Nrf2 expression	10 µM
Indy Tamayose et al. (2019)	Acellular	---	n/a	4,5-DCQA	DPPH	14.3 µM (IC_50_)
Tong et al. (2017)	Hep-G2 cells	Acrolein	1–5 µM	1,3-DCQA	DCF	1 µM
Topal et al. (2015)	Acellular	---	10–30 μg/mL	1,3-DCQA	DPPH	3.98 μg/mL = 7.7 µM (IC_50_)
Wang and Xiao (2019)	Mice	Acute lung injury via LPS	5, 10, 20 mg via i.p.	3,5-DCQA	MDA	5 mg
					SOD expression	10 mg
Xu et al. (2012)	Acellular	---	n/a	3,4-DCQA	DPPH	18.2 µM (EC_50_)
				3,5-DCQA		18 µM (EC_50_)
				4,5-DCQA		14.5 µM (EC_50_)
Zha et al. (2007)	Rat liver homogenate	Ascorbate-Fe^2+^	5–50 µM	3,5-DCQA	MDA	5 µM
Zheleva-Dimitrova et al. (2016)	Acellular	---	n/a	3,5-DCQA	DPPH	2.62 μg/mL = 5.1 µM (IC_50_)

PoDs representing IC_50_ or EC_50_ values are indicated, respectively. CAT, catalase, DCF, dichlorofluorescin, DPPH, 2,2-diphenyl-1-picrylhydrazyl, G6PD, glucose-6-phosphate dehydrogenase, GPX, glutathione peroxidase, GR, glutathione reductase, GSH, glutathione, HO-1, heme oxygenase, LPS, lipopolysaccharide, MDA, malondialdehyde, n/a: not available, TBHP, tert.-butylhydroperoxide.

#### 4.1.1 Scavenging of radicals

**FIGURE 4:**
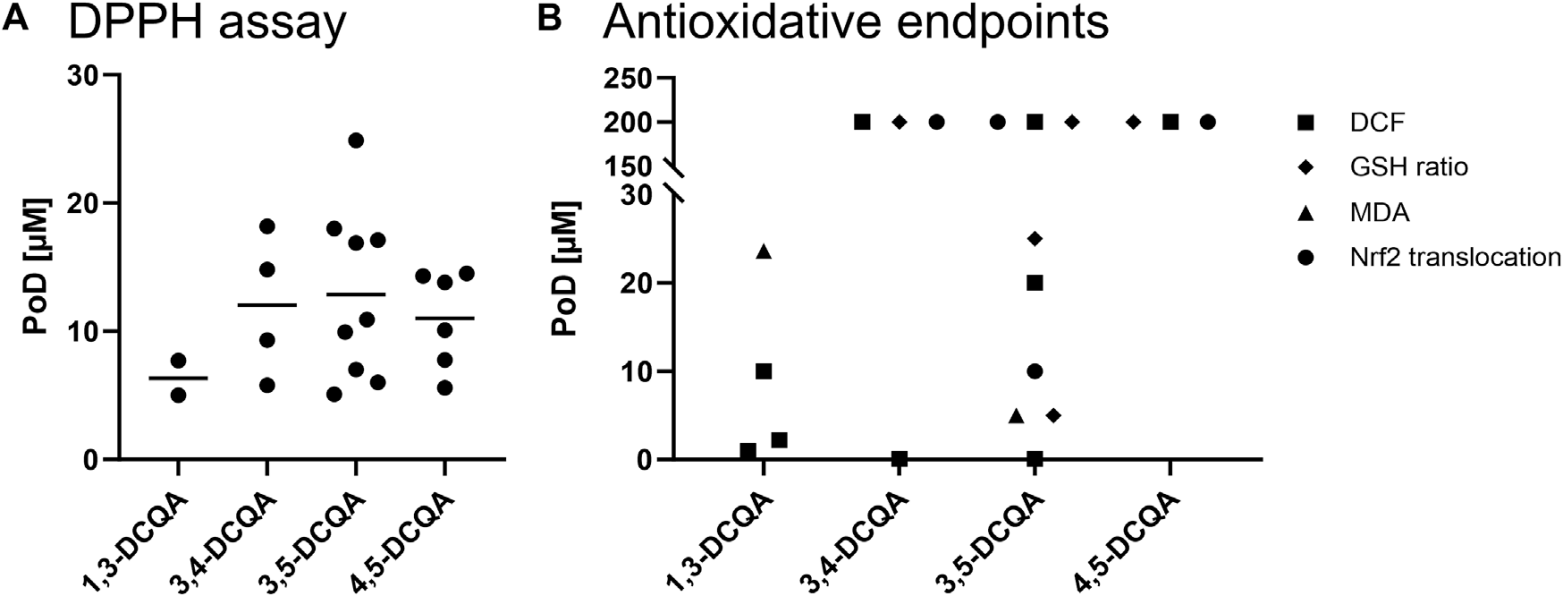
*In vitro* data on antioxidant properties of DCQAs across different studies. Data is displayed as a scatter plot where each dot represents a point of departure (PoD) obtained from different studies. **(A)** PoDs of applied acellular DPPH assays, the lines within the plot indicate the mean of the respective data. **(B)** PoDs on antioxidative endpoints investigated in cellular models. ■ DCF assay, ▲MDA assay, **♦** cellular GSH ratio, ● Nrf2 activation.

Various assays can evaluate the antioxidant potential on a cell-free basis, including 2,2-diphenyl-1-picrylhydrazyl (DPPH), 2,2′-azino-bis(3-ethylbenzothiazoline-6-sulfonic acid) (ABTS), and ferric ion reducing antioxidant power (FRAP) assays. All assays measure the potential of test items to capture free radicals. The DPPH assay was used most frequently to examine the scavenging of radicals by DCQAs. Using single substances, IC_50_ values for 1,3-DCQA in the range of 5–50 μM, for 3,4-DCQA in the range of 6–20 μM, for 3,5-DCQA in the range of 5.1–21 μM, and for 4,5-DCQA in the range of 5.6–15 µM were obtained (Psotová et al., 2003; Iwai et al., 2004; Park et al., 2009; Könczöl et al., 2012; Xu et al., 2012; Ma et al., 2015; Topal et al., 2015; Zheleva-Dimitrova et al., 2016; Bouratoua et al., 2018; Indy Tamayose et al., 2019; Kim C.-K. et al., 2022). When comparing the mean IC_50_ values of the evaluated data, there was no clear difference found between the isoforms 3,4-, 3,5-, and 4,5-DCQA, with respective mean IC_50_ values of 12, 13, and 11 µM (Figure 4A). However, 1,3-DCQA displayed a mean IC_50_ value of 6.35 µM, indicating a slightly higher antioxidative potential in this assay but only a sample size of n = 2. Besides studies testing single substances, various data is available showing a radical scavenging potential of extracts containing DCQAs (e.g., Paşayeva et al., 2022; Acquaviva et al., 2023; Zheleva-Dimitrova et al., 2023).

#### 4.1.2 Cellular oxidative stress response

##### 4.1.2.1 Intracellular ROS scavenging

The dichlorofluorescin (DCF) assay is a cell-based method used to investigate the scavenging of radicals in addition to cell-free assays. Within this assay, a fluorescent dye binds to free radicals inside cells if respective radicals are not detoxified beforehand by antioxidative substances or proteins. To determine scavenging of radicals, DCQAs as single substance were applied to different cell types of both, human and rodent origin together with a radical forming oxidant. These studies revealed PoDs in the range of 1–3 µM for 1,3-DCQA (Pérez-García et al., 2000; Pagano et al., 2016; Tong et al., 2017). The range of PoDs for 3,4- and 3,5-DCQA were quite large, spanning 0.01–200 µM (Kang et al., 2011; Kim et al., 2012; Liang and Kitts, 2018), and only one study was available that applied 4,5-DCQA with a PoD of 200 µM (Liang and Kitts, 2018). Due to the wide range of PoD values, a comparison across DCQA isoforms cannot be made (Figure 4B). It is noteworthy that the PoD of 200 µM detected by Liang and Kitts (2018) represents the lowest applied concentration. Therefore, it is possible that lower concentrations also induce an antioxidant effect detected by the DCF assay.

##### 4.1.2.2 Inhibition of lipid peroxidation

The inhibition of lipid peroxidation by oxidants determined by measurement of malondialdehyde (MDA) is an adverse process leading to cellular damage with subsequent pathologies. The detection of MDA was used to investigate the antioxidative potency of DCQAs mainly after applying a noxious substance such as tert-butylhydroperoxide (TBHP) or lipopolysaccharide (LPS). The inhibition of TBHP- or LPS- induced lipid peroxidation by DCQAs as single substance has been investigated (Figure 4B) in human hepatocytes and mitochondria as well as microsomes from rat liver homogenate (Gebhardt and Fausel, 1997; Psotová et al., 2003; Zha et al., 2007). Gebhardt and Fausel (1997) applied TBHP to primary hepatocytes to induce oxidative stress. Subsequently, selected compounds, including 1,3-DCQA, were applied to the cell culture to investigate the antioxidant activity of the test items. The study found that 1,3-DCQA had an EC_50_ value of 23.6 µM, which was the lowest compared to caffeic acid, chlorogenic acid, and cynaroside with EC_50_ values of 45, 35.3, and 62.4 µM, respectively (Gebhardt and Fausel, 1997). Psotová et al. (2003) assessed the antioxidant activity by investigating TBHP-induced lipid peroxidation in the mitochondrial fraction of rat liver homogenate dependent on the co-incubation with phenolic substances. The results indicated that 1,3-DCQA exhibited an IC_50_ value of 1.96 mM, whereas protocatechuic, caffeic, rosmarinic, ferulic and chlorogenic acid revealed IC_50_ values of > 2, 0.59, 0.09, > 2, and 2.37 mM, respectively (Psotová et al., 2003). Lipid peroxidation in microsomes derived from rat liver homogenate following ascorbate-Fe^2+^-induced oxidative stress was investigated for its prevention by 3,5-DCQA. Results indicated a significant and dose-dependent decrease in MDA levels already at 5 µM 3,5-DCQA (Zha et al., 2007). Regarding this endpoint, 3,5-DCQA seemed to be more active than 1,3-DCQA. Besides *in vitro*, Wang and Xiao (2019) induced acute lung injury to mice using LPS and treated this model with 5, 10, and 20 mg of 3,5-DCQA via intraperitoneal injection. Within this study, MDA level in lung tissue of the acute lung injury model strongly increased and dose-dependently decreased after application of 3,5-DCQA. The decrease was significant after applying 5 mg and returned to basal MDA levels at 20 mg 3,5-DCQA (Wang and Xiao, 2019). In addition to testing single substances, DCQAs containing extracts were tested *in vivo* for their effect on lipid peroxidation by determining MDA concentration (Yin et al., 2018; Li et al., 2019). This strengthened the observation, that DCQA-rich extracts induce the antioxidant activity *in vivo*.

##### 4.1.2.3 Induction of antioxidative protein

Glutathione (GSH) is a peptide with well-established antioxidant properties in cells. It is present in two forms: the reduced form, GSH, and the oxidized form, glutathione disulfide (GSSG). The ratio of GSH to GSSG is a crucial parameter in assessing cellular oxidative stress levels (Franco and Cidlowski, 2012). The impact of DCQAs as single substance on the cellular GSH level has been assessed in various studies (Figure 4B) *in vitro* (Kim et al., 2005; Kang et al., 2011; Liang and Kitts, 2018). Within these studies, the cellular GSH content of cells derived from liver (HepG2), colon (Caco-2), and bone tissue (SH-SY5Y) was determined after an oxidative stimulus and subsequent DCQA incubation. The PoDs obtained were determined to be 5 µM (Kang et al., 2011), 25 µM (Kim et al., 2005), and 200 µM (Liang and Kitts, 2018) for 3,5-DCQA as well as 200 µM for 3,4- and 4,5-DCQA (Liang and Kitts, 2018). It must be noted, however, that the PoD of 200 µM across these studies is due to the circumstance of 200 µM being the lowest applied concentration in the respective study by Liang and Kitts (2018). Hence, the range of PoDs from 5–200 µM for 3,5-DCQA can be explained by the choice of concentrations in one specific study. Due to the wide range of applied concentrations and limited data on 1,3-, 3,4-, and 4,5-DCQA no comparison on the activity of DCQA isoforms can be made. In addition to *in vitro* experiments, 4,5-DCQA was administered orally at doses of 5, 10, and 20 mg/kg bw to mice of a NASH model over the period of 4 weeks. The GSH level in the liver decreased significantly in the NASH model compared to a normal mice group, whereas the additional treatment with 4,5-DCQA resulted in a dose-dependent increase of liver GSH starting at 5 mg/kg bw. At 20 mg/kg bw the basal GSH level was nearly reconstituted (Liu et al., 2019b). Even though this model does not represent a lung-specific disease model, the authors suggest a similar effect during inflammation of the respiratory tract after systemic application of DCQAs. Furthermore, the effect of DCQA-containing extract on cellular GSH levels was determined in oxidative-stimulated keratinocytes (HaCaT) and hepatocytes derived from both human and rats origins (Gebhardt and Fausel, 1997; Yin et al., 2018; Li et al., 2019; Gao et al., 2021; Zheleva-Dimitrova et al., 2023). Overall, the *in vitro* and *in vivo* data indicates that DCQAs reconstitutes the cellular GSH level after applying an oxidative trigger.

Another mechanism of antioxidative defense is the expression of antioxidative proteins such as superperoxide dismutase (SOD), catalase (CAT), or heme oxygenase (HO-1). The protein levels of different antioxidant acting proteins have been evaluated *in vitro* and *in vivo* applying single substances as well as DCQA-containing extracts. Cao and colleagues (2017) applied 100 µM 3,5-DCQA to kidney epithelial cells (Vero cells) after infecting the cell culture with EV71 virus. Viral inoculation resulted in a decreased expression of GSH metabolic enzymes, i.e., glutathione reductase (GR), glutathione peroxidase (GPX), and glucose-6-phosphate dehydrogenase (G6PD) (Rahman et al., 1999). Treating the cells with 100 µM 3,5-DCQA returned basal protein levels of GSH homeostasis-related proteins (Cao et al., 2017). In addition, the impact of 3,5-DCQA on the expression of HO-1 on 3T3-L1 cells after treatment with a hormonal cocktail (MDI) was investigated. Expression of HO-1 on a protein level was elevated after incubation with 10 µM 3,5-DCQA compared to a MDI-treated control (Raineri et al., 2021). Regarding *in vivo* studies, within a mouse model of acute lung injury induced by LPS applying 3,5-DCQA by intraperitoneal injection at doses of 5, 10 or 20 mg SOD levels in the lung were clearly decreased within the acute lung injury model and was elevated statistically significant after treatment of 10 and 20 mg 3,5-DCQA (Wang and Xiao, 2019). The effect on antioxidative proteins (SOD, GPX, CAT) was also investigated using DCQA-containing extracts *in vivo* (Kwon et al., 2019; Li et al., 2019). Taken together, these data suggest that DCQAs induce antioxidant activity not only through their ability to scavenge free radicals, but also by inducing the expression of proteins critical for cellular antioxidant defense mechanisms. As summarized by Zuo and Wijegunawardana, ROS play an important role in the pathogenesis of respiratory diseases such as COPD, asthma, and inflammation-associated disease as their further enhance ROS production (Zuo and Wijegunawardana, 2021). Therefore, not only scavenging of ROS by DCQA but also the activation antioxidative stress response can have a beneficial impact in the treatment of respiratory diseases.

#### 4.1.3 Antioxidative pathway signaling

**FIGURE 5:**
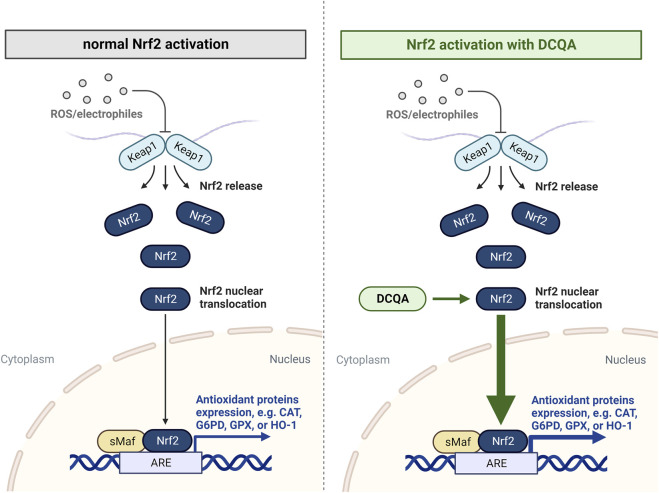
Nrf2/Keap1 pathway activation under normal conditions and after treatment with dicaffeoylquinic acids. Keap1 regulates the activity of the transcription factor Nrf2. Under conditions of oxidative stress, Nrf2 is released from Keap1 and translocates to the nucleus, where it activates the expression of genes encoding antioxidant enzymes. Data indicates an enhanced nuclear translocation of Nrf2 after treatment with DCQAs. ARE: antioxidant response element; DCQA: dicaffeoylquinic acid; CAT: catalase; G6PD: glucose-6-phosphate dehydrogenase; GPX: glutathione peroxidase; HO-1: heme oxygenase-1 (Figure created using BioRender, Toronto, ON, Canada).

The Nrf2/Keap1 pathway is a key player within cellular oxidative defense being activated subsequent to toxic and oxidative stress. It acts via the expression of genes associated to oxidative stress response and drug detoxification as well as other cellular processes, including inflammation (He et al., 2020). Therefore, activation of this signaling pathway after cellular stress is crucial for maintaining cellular redox balance and to enhance cell survival under exogenous stress (Figure 5).

The ability of DCQAs to activate the Nrf2 pathway was mainly assessed investigating nuclear translocation of Nrf2 (Figure 4B). Cao et al. (2010) used primary rat astrocytes to evaluate Nrf2 activation by 1,5-DCQA. At a concentration of 50 µM a statistically significant increase in Nrf2 translocation to the nucleus was observed (Cao et al., 2010). A similar observation was made after treating Caco-2 cells with 200 µM each of 3,4-, 3,5-, and 4,5-DCQA, which represents the lowest applied concentration in the respective study (Liang and Kitts, 2018). Furthermore, Raineri et al. (2021) observed an enhanced Nrf2 protein expression after 24 h treatment with 10 µM 3,5-DCQA in 3T3-L1 cells (Raineri et al., 2021). As stated previously for GSH assessment, a comparison between on the Nrf2 translocation of DCQA isoforms cannot be made due the scarce data on 1,3-, 3,4-, and 4,5-DCQA. In addition to *in vitro* studies, nuclear translocation of Nrf2 in a NASH mice model after treatment with 5 mg/kg bw 4,5-DCQA was observed, whereas the untreated NASH mice as well as normal control mice treated with 4,5-DCQA did not show nuclear translocation, respectively (Liu et al., 2019b). This observation has not yet been investigated within a disease model associated with the respiratory tract, however, the authors suggest this effect can be observed in other target tissues since it is an unspecific reaction. All this data indicates the potential of DCQAs to activate the essential antioxidative Nrf2/Keap1 pathway *in vitro* and *in vivo*. This is further strengthened by the above described upregulation of antioxidative proteins, which in addition represent Nrf2 target genes, i.e., *CAT, G6PD, GR, GPX, H O -1*, and *SOD* (Baird and Yamamoto, 2020; He et al., 2020). It was shown that Nrf2 deactivation results in high susceptibility and increased disease severity in different respiratory disease models, e.g., COPD, asthma, respiratory infections, and idiopathic pulmonary fibrosis. Accordingly, the activation of this pathway results in a protection against various respiratory diseases (Liu et al., 2019a; Mizumura et al., 2020). This clearly shows that DCQA-induced activation of Nrf2 can contribute beneficially in the treatment of respiratory diseases.

### 4.2 Anti-inflammatory properties

Several mechanisms can contribute to an anti-inflammatory effect induced by drugs including regulation of the expression of inflammation mediators and cytokines, inhibition of certain proteins or pathways as well as modulation of the immune system, e.g., by blocking specific receptors. The next chapters will emphasize the anti-inflammatory properties of DCQAs which are also summarized in Table 4.

**TABLE 4:** Summary of studies investigating anti-inflammatory properties of dicaffeoylquinic acids (DCQA) *in vitro* or *in vivo* listing relevant endpoints and point of departures (PoD), which indicate a significant effect on the respective endpoint.

Reference	Model	Trigger	Applied Dose/Concentration	DCQA isoform	Endpoint	PoD
Chen et al. (2015)	RAW264.7 cells	LPS	25 µM	3,5-DCQA	NO production	25 µM
			25 µM	4,5-DCQA		25 µM
			12.5 µM	3,4-DCQA		12.5 µM
Chen et al. (2016)	Mice	Acute lung injury via LPS	25. 50 mg/kg bw, i.p.	3,5-DCQA	TNFα/IL-6 protein secretion in BALF	25 mg/kg bw
Jang et al. (2021)	RAW264.7 cells	LPS	1, 2, 4 µM	4,5-DCQA	TNFα protein expression, NO production, p65 translocation, p38/JNK phosphorylation	4 µM
					IL-6/iNOS/COX-2/PGE_2_ protein expression, IκBα phosphorylation	2 µM
Jang et al. (2022)	Rat primary chondrocytes	IL-1β	10, 20, 40 µM	4,5-DCQA	TNFα/iNOS/COX-2 protein expression, NO production	10 µM
					PGE_2_ protein expression, p65 translocation	20 µM
					IκBα phosphorylation	40 µM
Kim et al. (2020)	RAW264.7 cells	LPS	n/a	3,4-DCQA	NO production	7.95 µM (IC_50_)
			12,5–50 µM		IL-6 protein expression	25 µM
			12,5–50 µM		TNFα protein expression	50 µM
Kim et al. (2022b)	EA.hy926 cells	LPS	1 & 5 µM	1,3-DCQA	*IL-1b* expression, p38 phosphorylation	1 µM
					p65 translocation	5 µM
Li et al. (2013a)	Mice macrophages	LPS	50 & 190 µM	3,5-DCQA	IL-6 protein expression	50 µM
				3,4-DCQA		
				4,5-DCQA		
Li et al. (2022)	RAW264.7 cells	LPS	3.125–100 µM	3,5-DCQA	NO production	3.125 µM
Noh et al. (2022)	BMDMs	LPS	10, 25, 50 µM	3,5-DCQA	IL-1β protein expression	10 µM
Park et al. (2022)	Mice	Complete Freund Adjuvant	10, 30 mg/kg bw, p.o.	3,5-DCQA	STAT3 phosphorylation	30 mg/kg bw
Puangpraphant et al. (2011)	RAW264.7 cells	LPS	1–200 µM	4,5-DCQA	iNOS protein expression	50 µM
					COX-2 protein expression	200 µM
Tang et al. (2023)	J774A.1 cells	LPS + IFNγ	5, 100, 200 µM	3,5-DCQA	STAT3 phosphorylation	50 µM
					*IL-1b/TNFa/IL-6/iNOS* expression, p65/IκBα phosphorylation	100 µM
	RAW264.7 cells	LPS + IFNγ	5, 100, 200 µM	3,5-DCQA	*IL-6* expression, p65/IκBα/STAT3 phosphorylation	100 µM
					*IL-1b/iNOS* expression	200 µM
Tian et al. (2020a)	RAW264.7 cells	LPS	20, 40, 80 µM	3,4-DCQA	NO production PGE_2_ protein expression	80 µM 20 µM
				3,5-DCQA	NO production PGE_2_ protein expression	40 µM 20 µM
				4,5-DCQA	NO production PGE_2_ protein expression	40 µM 20 µM
Wang and Xiao (2019)	Mice	Acute lung injury via LPS	5, 10, 20 mg, i.p.	3,5-DCQA	IL-1β/IL-6/CCL2/iNOS/COX-2/NLRP3 protein expression (lung), TNFα protein expression in BALF, p65 phosphorylation in lung tissue	5 mg
Wu et al. (2022)	BMDMs	LPS	290 µM	1,3-DCQA	*IL-1b/IL-6/iNOS* expression	290 µM
	Mice	MSU (Na-Urate)	25 mg/kg bw, i.p.	1,3-DCQA	NLRP3 protein expression	25 mg/kg bw
Xia et al. (2014)	Human coronary artery smooth muscle cells	Cytokine mix	10 µM	1,3-DCQA	*iNOS* expression, iNOS protein expression	10 µM
Yang et al. (2023)	MH7a cells	TNFα	20 µM	3,5-DCQA	*IL-1b/IL-17A* expression, IL-1β/IL-17A protein expression	20 µM
	RAW264.7 cells	LPS	1, 20, 50 µM	3,5-DCQA	IL-17A protein expression	20 µM
					ERK phosphorylation	1 µM
Zheng et al. (2022)	BMDMs	Ox-LDL	10 µM		*IL-1b/iNOS* expression, IL-1β/iNOS/COX-2 protein expression, p65/IκBα phosphorylation	10 µM

BALF, bronchoalveolar lavage fluid; BMDM, bone marrow-derived macrophages; iNOS, inducible nitric oxide synthase; JNK, c-Jun NH2-terminal kinases; LPS, lipopolysaccharide; PGE_2_, prostaglandin E2; TBHP, tert.-butylhydroperoxide.

#### 4.2.1 Regulation of inflammation mediators and cytokines

Cytokines are described as rather small, secreted proteins inducing and controlling the immune response. After lung injury or infection they control the respective response possibly resulting in clearance of the disease, repair of tissue and lastly return to homeostasis (Atamas et al., 2013). The impact of DCQAs on several cytokines and mediators, which are involved in the pathogeneses of respiratory diseases, was evaluated.

##### 4.2.1.1 Downregulation of IL-1β

**FIGURE 6:**
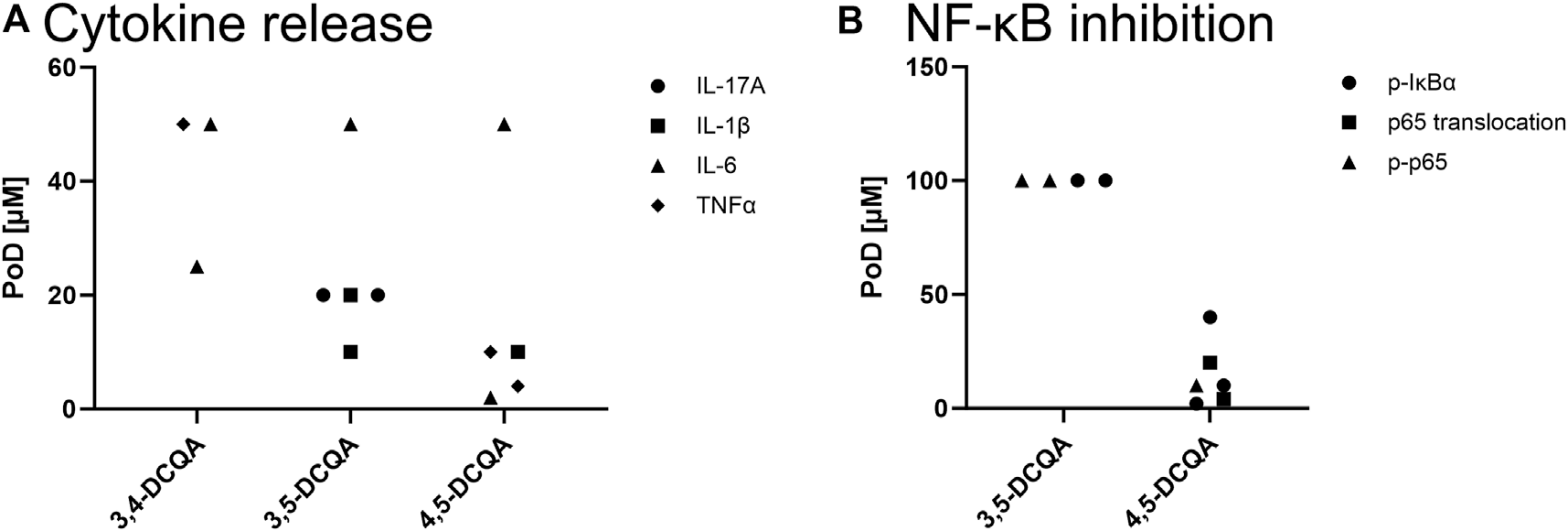
*In vitro* data on anti-inflammatory properties of DCQAs across different studies. Data is displayed as a scatter plot where each dot represents a point of departure (PoD) obtained from different studies. **(A)** PoDs regarding the inhibition of cytokine secretion on a protein level. ● IL-17A release, ▲ IL-6 release, ■ IL-1β release, **♦** TNFα release. **(B)** PoDs on the inhibition of the NF-κB activation. ● Phosphorylation of IκBα, ■ p65 translocation in the nucleus, ▲ phosphorylation of p65.

IL-1β represents an IL-1 type cytokine and therefore a major mediator of innate immune reactions. Indicated as pro-inflammatory cytokine, its activation results in cellular cascades via NF-κB and MAPK pathways and subsequent expression of various target genes, e.g., *IL-6, COX-2* or *IL-1b* (Weber et al., 2010). The alleviation of an IL-1β-mediated inflammatory response by DCQAs has been evaluated applying 1,3-, 3,5-, and 4,5- DCQA to several rodent- (RAW264.7, BMDMs, J774A.1, BV2) and human-based (EA.hy926, MH7A) cells on a transcriptional and protein level after incubation with stimuli, i.e., LPS, IFN-γ, TNFα, or ox-LDL. mRNA expression of IL-1β after stimuli was decreased by all applied DCQAs with PoDs ranging from 1–290 µM for 1,3-DCQA, of 10–200 µM for 3,5-DCQA, and of 10 µM for 4,5-DCQA (Kim D. B. et al., 2022; Park et al., 2022; Wu et al., 2022; Zheng et al., 2022; Tang et al., 2023; Yang et al., 2023). Comparing this data, the lowest observed PoD is similar for all DCQAs, however a relatively wide range for 1,3- and 3,5-DCQA is apparent. Expression of *IL-1b* after induced inflammation was verified *in vitro* on a protein level (Figure 6A) in MH7A cells and bone marrow-derived macrophages (BMDMs) for 3,5-DCQA (PoDs: 10–20 µM) and 4,5-DCQA (PoD: 10 µM) after incubation with LPS or ox-LDL (Noh et al., 2022; Zheng et al., 2022; Yang et al., 2023). Here, no relevant difference in PoDs between DCQAs and thus no difference in biological activity, was observed. A decrease of IL-1β after a pro-inflammatory stimuli was also confirmed *in vivo* for 3,5-DCQA. In a lung specific model, a decrease in IL-1β secretion after intraperitoneal injection of 5 mg to an acute lung injury model in mice was observed (Wang and Xiao, 2019; Tang et al., 2023). This further indicates the anti-inflammatory properties of DCQAs *in vitro* and *in vivo*.

##### 4.2.1.2 Regulation of IL-17A/TNFα

IL-17A is involved in various pathological outcomes, including acute and chronic respiratory diseases (Gurczynski and Moore, 2018). Yang et al. (2023) observed a decrease of the IL-17A protein level in MH7A and RAW264.7 cells after a pro-inflammatory trigger with TNFα or LPS, respectively, and subsequent 3,5-DCQA treatment with 20 µM and higher (Yang et al., 2023). Furthermore, IL-17 is able to induce TNFα in epithelial, endothelial, and fibroblastic cells. The lung is one of many organs which is affected by TNFα within inflammatory diseases, e.g., chronic bronchitis, COPD, and asthma. TNFα-dependent inflammation is supposed to be based on deregulation of leukocytes and lymphocytes recruitment as well as depletion of the cellular antioxidant GSH and hence the induction of oxidative stress (Mukhopadhyay et al., 2006). Therefore, TNFα secretion is one parameter to evaluate the anti-inflammatory potential of DCQAs. The impact of DCQAs on TNFα within stimulated cell systems has been investigated in various studies (Figure 6A). 3,4- and 4,5-DCQA were applied to stimulated RAW264.7 resulting in a decrease of TNFα secretion (Kim et al., 2020; Jang et al., 2021). Furthermore, primary rat chondrocytes were treated with 4,5-DCQA, respectively, resulting in a similar observation of decreased TNFα protein level (Jang et al., 2022; Park et al., 2022). Taken together, PoDs of 50 μM and 4–10 µM were determined for 3,4- and 4,5-DCQA, respectively. This indicates, that 4,5-DCQA seems to obtain a higher biological activity regarding inhibition of TNFα secretion compared to 3,4- and 3,5-DCQA. An impact of 3,5- and 4,5-DCQA on the TNFα expression was also observed within *in vivo* studies. In more detail, TNFα protein expression was markedly decreased in lung tissue as well as in bronchoalveolar lavage fluid (BALF) of an acute lung injury mice model after intraperitoneal injection of 5 mg and 25 mg/kg bw 3,5-DCQA, respectively (Chen et al., 2016; Wang and Xiao, 2019). Thus, *in vivo* data confirms the potential of DCQAs to inhibit TNFα, and therefore an important pro-inflammatory mediator.

##### 4.2.1.3 Impact on IL-6 regulation

IL-6 represents another pro-inflammatory cytokine with an important role in respiratory diseases associated with inflammation but also in the pathogenesis of asthma and potentially COPD (Rincon and Irvin, 2012). It binds to its receptor IL-6R which subsequently associates with gp130 resulting in an activation of specific JAK tyrosine kinases, following phosphorylation, and activation of transcription factors such as STAT3. Furthermore, IL-6 dependent activation of C/EBPβ via the MAPK pathway is known (Rincon and Irvin, 2012). On a transcriptional level, reduction of LPS-induced *IL-6* expression was observed *in vitro* after treatment with 1,3- and 3,5-DCQA at PoDs of 290 µM and 100 μM, respectively (Park et al., 2022; Wu et al., 2022; Tang et al., 2023). Comparing this data, no relevant difference across cell systems and DCQAs were obvious considering that Wu and colleagues (2022) only used one concentration of 290 µM which explains the higher PoD of 1,3-DCQA. IL-6 expression was further evaluated *in vitro* on a protein level resulting in a similar observation of decreased IL-6 levels in inflammatory cell models after DCQAs incubation. In more detail, PoDs for reduced IL-6 expression ranging between 25–50 µM for 3,4-DCQA, at 50 µM for 3,5-DCQA, and a range of 2–50 µM for 4,5-DCQA were obtained (Li Y. et al., 2013; Kim et al., 2020; Jang et al., 2021; Park et al., 2022). The comparison of PoDs suggests that protein expression seems to be more sensitive than transcriptional assessment of IL-6 and further that 4,5-DCQA is suggested to be biologically slightly more active than 3,4- and 3,5-DCQA (Figure 6A). IL-6 secretion was also investigated *in vivo* after treatment of inflammation models with 3,5-DCQA. Most importantly, two studies investigated IL-6 secretion in lung tissue and BALF after intraperitoneal injection of 3,5-DCQA in a LPS-induced acute lung injury model in mice. While the IL-6 protein level in lung tissue was already significantly reduced applying 5 mg 3,5-DCQA (Wang and Xiao, 2019), the IL-6 level in BALF was markedly decreased at a dose of 25 mg/kg bw (Chen et al., 2016). The *in vivo* data confirms the anti-inflammatory properties in regard of inhibition of IL-6 secretion observed *in vitro*. The observation of reduced IL-6 in stimulated cell and animal models treated with DCQAs is in line with the observation on the downstream IL-6-dependent activation of STAT3. Treatment with 3,5-DCQA resulted in a decrease in phosphorylated and thus activated STAT3 in stimulated rodent cells at concentrations as low as 100 µM (Park et al., 2022; Tang et al., 2023). Furthermore, 3,5-DCQA at a dose of 30 mg/kg bw reduced the protein level of phosphorylated STAT3 in the spinal cord tissue of a complete Freund’s adjuvant-induced inflammatory model in mice (Park et al., 2022).

##### 4.2.1.4 Evaluation of impact in CCL2

Lastly, the protein level of the chemokine CCL2, which is suggested to be involved in regulation of neutrophilic lung inflammation (Mercer et al., 2014), was evaluated *in vivo*. Wang and Xiao (2019) applied 3,5-DCQA via intraperitoneal injection to an acute lung injury model in mice induced by LPS and observed a decrease of CCL2 secretion after an administration of 5 mg 3,5-DCQA i. p. (Wang and Xiao, 2019).

##### 4.2.1.5 Inhibition pro-inflammatory protein expression

Besides the inhibition of cytokines, DCQAs impact further inflammatory mediators, such as iNOS expression, NO production, COX-2 expression, and PGE_2_ expression. Under physiological conditions the nitric oxide synthase (NOS) produces endogenous NO via a constitutive NOS isoform. During inflammatory processes, however, an inducible NOS (iNOS) isoform is expressed and results in an excess of NO, which can regulate expression of certain cytokines and chemokines (Speyer et al., 2003). An enhanced iNOS expression has been associated with several inflammatory diseases of the lung, e.g., COPD, asthma, and acute respiratory distress syndrome (Huang et al., 2015). The impact of DCQAs on iNOS expression on a transcriptional and protein level within inflammatory triggered cell models has mainly been evaluated applying rodent cell models. mRNA expression was decreased within inflammatory BMDMs, RAW264.7, and J77A.1 cell models after incubation with DCQAs with PoD of 290 µM for 1,3-DCQA (Wu et al., 2022), ranging between 100–200 µM for 3,5-DCQA (Tang et al., 2023), and of 10 µM for 4,5-DCQA (Zheng et al., 2022). On a protein level inflammatory models of RAW264.7 cells, primary rat chondrocytes, and BMDMs were treated with 4,5-DCQA resulting in PoDs of a range between 2–200 μM, respectively (Puangpraphant et al., 2011; Jang et al., 2021; Jang et al., 2022; Park et al., 2022; Zheng et al., 2022). Xia et al. (2014) assessed the impact of 1,3-DCQA treatment in iNOS expression in a cytokine-induced inflammatory model of human coronary artery smooth muscle cells observing a decrease of induced iNOS expression at 10 µM for both, transcriptional and protein expression (Xia et al., 2014). Taken this information together, it is indicated that protein expression is more sensitive than transcription of iNOS without a relevant difference of biological activity between evaluated DCQAs. The expression of iNOS was also evaluated *in vivo* on a protein level. Here, the protein level in lung was decreased after administration of 5 mg 3,5-DCQA applied via intraperitoneal injection to an acute lung injury model in mice (Wang and Xiao, 2019; Jang et al., 2021). The *in vivo* data emphasizes potential to inhibit iNOS observed *in vitro*.

Besides expression of iNOS, the downstream endpoint NO production has been assessed in numerous studies. Excessively produced NO by iNOS is indicated to be involved in constriction, inflammation and remodeling of the lung observed in asthma, hence are associated with adverse effects (Prado et al., 2011). Therefore, reduction of increased NO is suggested to be beneficial for inflammatory respiratory diseases. NO production *in vitro* was mainly assessed in LPS-stimulated RAW264.7 cells with PoDs ranging from 8–80 µM for 3,4-DCQA, 3–40 µM for 3,5-DCQA, and 4–40 µM for 4,5- DCQA (Chen et al., 2015; Tian D. et al., 2020; Kim et al., 2020; Jang et al., 2021; Li et al., 2022). Within IL-1β-stimulated primary rat chondrocytes a PoD of 10 µM was observed after 4,5-DCQA treatment (Jang et al., 2022). Taken together, no isoform-dependent biological activity was observed for this endpoint.

COX-2 is an inducible isoform of cyclooxygenase which catalyzes the cyclooxygenation of arachidonic acid to prostaglandin G_2_ and subsequently H_2_. The promotor region of *COX-2* contains various transcriptional regulatory elements such as the cAMP response element (CRE) or for NF-κB (Park and Christman, 2006). Hence, an induction or repression of COX-2 can be related to an activation or inhibition of the NF-κB signaling pathway. Protein expression of COX-2 was evaluated *in vitro* in various studies, mainly applying RAW264.7, BMDMs but also in BV2 cells and primary chondrocytes derived from rats. All cell models were first treated with a pro-inflammatory trigger, i.e., LPS, IL-1β, or ox-LDL and subsequently with 3,5- or 4,5-DCQA as single substance. In all test systems a decrease of COX-2 protein expression compared to the effects of sole inflammation-inducing agent was apparent with PoDs ranging between 2 and 20 µM for 4,5-DCQA (Puangpraphant et al., 2011; Jang et al., 2021; Jang et al., 2022; Park et al., 2022; Zheng et al., 2022). Comparing protein expression PoDs and biological activity across DCQAs, no relevant difference is apparent. Data on COX-2 expression was also available for an *in vivo* study. Wang and Xiao (2019) observed a reduction of COX-2 protein level in lung tissue after applying 5 mg 3,5-DCQA intraperitoneally to an acute lung injury model in mice. Therefore, the *in vitro* determined amelioration of COX-2 induction after inflammatory stimuli was confirmed *in vivo* for the two isoforms tested.

The same pattern as for COX-2 has been observed for a reaction product of COX-2, prostaglandin E_2_ (PGE_2_). Within LPS- or IL-1β-stimulated rodent cell models 3,4-, 3,5-, and 4,5-DCQA reduced the PGE_2_ level with PoDs ranging without differences regarding the isoforms from 2–20 µM (Tian K. et al., 2020; Jang et al., 2021; Jang et al., 2022).

##### 4.2.1.6 Evaluation of DCQA-mediated impact on respiratory diseases

The occurrence of inflammation is common in broad span of respiratory diseases, e.g., acute/chronic bronchitis, asthma, or COPD. It was shown that INF-α, INF-γ, TNFα, IL-10 and IL-6 were enhanced in plasma after influenza virus infection (Kaiser et al., 2001). More importantly, IL-6 and TNFα plasma and nasopharyngeal lavage level correlated with symptom scores as well as temperature values (Kaiser et al., 2001). Hence, the reduction of such cytokines by DCQAs can strongly contribute to the reduction of symptoms and play an important role in the treatment of inflammation-related respiratory diseases. Besides, increase of the cytokine IL-17A results in enhanced IL-6 levels, which further increases MUC5AC and MUC5B protein levels (Kim and Criner, 2013). These proteins play a crucial role in the mucus and enhanced expression can be seen as increased mucus production. Hence, decreasing IL-17A as well as IL-6 by DCQAs is a valid treatment option for symptom relief. Furthermore, IL-17 and IL-6 play an important role in Th2-low asthma and therefore a co-treatment with DCQAs might also be beneficial in reducing inflammation during this severe respiratory disease (Ramakrishnan et al., 2019; Habib et al., 2022). As previously stated the induction of iNOS expression correlates with several inflammatory diseases of the lung, e.g., COPD, asthma, and acute respiratory distress syndrome (Huang et al., 2015). Furthermore iNOS-triggered NO inducues mucus secretion in allergic asthma and also results in enhanced ROS levels (Lee et al., 2021). Therefore, reduction of iNOS expression, as observed after treatment with DCQAs, can result in a beneficial effect in treatment of respiratory diseases. Last but not least, COX-2 is expressed in response to various pro-inflammatory cytokines and mediators. Hence, induction of COX-2 is associated with the pathology of inflammation-related respiratory diseases (Rumzhum and Ammit, 2016). Again, the treatment with DCQAs can contribute in a positive way for respective respiratory diseases.

#### 4.2.2 Immuno-modulating properties

Immunomodulation can be described as the property of a drug to shift the balance of the activity of immune cells. This effect can be both activating and suppressive. Very briefly, different cell types can be involved in an immunogenic response: different granulocytes, macrophages, dendritic cells, monocytes, mast cells, and various differentiated lymphocytes (Zebeaman et al., 2023). Within this chapter the potential of DCQAs to impact immune cells will be discussed, even though data is, so far, scarce.

Tatefuji et al. (1996) investigated the effect of 3,4-, 3,5-, and 4,5-DCQA on spreading and mobility of isolated murine macrophages. Both, spreading and mobility, was increased after applying the investigated DCQAs without revealing an isoform-dependent difference (Tatefuji et al., 1996). The result of this study can be interpreted as an activation of macrophages in a not stimulated, i.e., non-inflammatory, environment, thus DCQAs acting as immune-supporting substances. Additionally to this study, an immune-suppressive effect by 1,3-DCQA at roughly 200 µM was described *in vitro* via the blocking of CD28, a T-cell receptor that regulates CD-28-dependent IL-2 expression as well as being crucial for T-cell activation (Dong et al., 2006). In *in vivo* studies, a downregulation of neutrophils was observed in a BALF analysis of an acute lung injury model in mice after treatment with 25 mg/kg bw intraperitoneal injected 3,5-DCQA (Chen et al., 2016). In addition, the number of leucocytes was reduced in an acute airway inflammation mice model induced by ammonia liquor after oral administration of 20 mg/kg bw 3,4-, 3,5-, or 4,5-DCQA for 3 days (Wu et al., 2015). Even though all applied DCQAs reduced the number of leucocytes significantly, 3,4-DCQA obtained the highest biological activity in this study followed by 4,5- and 3,5- DCQA. Taken together, both studies showed the potential of DCQAs to downregulate inflammatory cells during an inflammation *in vivo*.

Taken together, immunomodulation by DCQAs is not sufficiently depicted in available studies. However, the potential to activate macrophages and to downregulate immune cells, especially during inflammation *in vivo*, indicates a potential immune-modulating property of DCQAs.

#### 4.2.3 Inflammation-related pathway signaling

##### 4.2.3.1 Deactivation of NF-κB pathway

**FIGURE 7:**
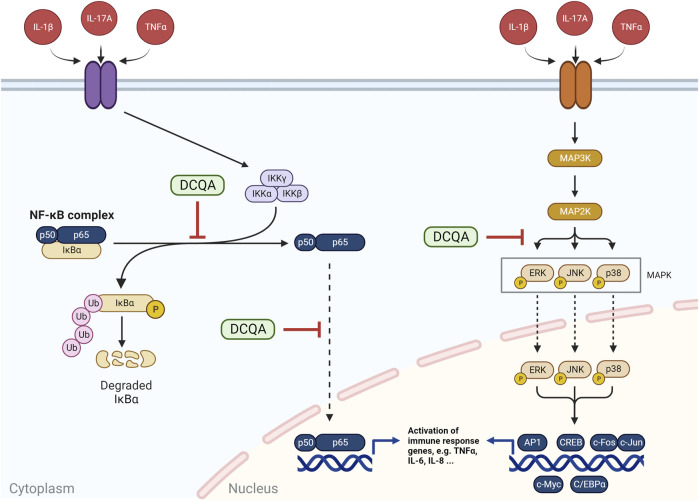
Schematic representation of the NF-κB and MAPK signaling pathways and the impact of DCQA on them. NF-κB represents a complex of p50 and p65, which interacts with IκBα. The latter is phosphorylated and ubiquitinated by an IKK complex after stimulation, resulting in an activation of NF-κB with subsequent p50 and p65 translocation into the nucleus and respective transcriptional activity. The MAPK (mitogen-activated protein kinase) pathway is a multi-tiered pathway with subsequent steps of phosphorylation-dependent activation from MAP3Ks to MAP2Ks and lastly MAPKs. ERK, JNK, and p38 represent MAPKs, which translocate into the nucleus and activate several transcription factors, e.g., AP-1, CREB, c-Myc, ord C/EBPα. DCQAs are suggested to inhibit the phosphorylation and ubiquitination of IκBα as well as the translocation of p65 and therefore NF-κB activation as well as the phosphorylation of MAPKs (Figure created using BioRender, Toronto, ON, Canada).

The NF-κB pathway is supposed to play an important part within the pathogenesis of inflammatory diseases. It can be activated by a variety of stimuli such as cytokines, oxidative stress, and UV radiation. It obtains numerous downstream targets, which includes cytokine expression. Evidence on the role of NF-κB signaling pathway in inflammation associated respiratory diseases, e.g., asthma and COPD, is convincing, therefore an impact on this pathway can be a gate opener for the use as therapeutic substance within these diseases (Edwards et al., 2009). As a brief summary, activation of this pathway includes phosphorylation of IκBα by IKKα with subsequently ubiquination of IκBα (Figure 7). This process results in a release of the NF-κB subunits p50/p65 from IκBα followed by their translocation in the nucleus and respective transcriptional activity (Edwards et al., 2009). The impact of DCQAs on the NF-κB pathway subsequent to its activation has been investigated *in vitro* on several levels (Figure 6B). Tang et al. (2023) evaluated the impact of 3,5-DCQA on the phosphorylation of IκBα in J774A.1 and RAW264.7 cells after stimulation with LPS and IFN-γ. Hereby, a decrease of IκBα phosphorylation after 3,5-DCQA treatment was observed at 100 µM. Furthermore, the same observation was made for 4,5-DCQA after treatment of rodent macrophages with pro-inflammatory triggers at PoDs of 2 and 10 µM (Jang et al., 2021; Zheng et al., 2022). In addition, IL-1β-stimulated and 4,5-DCQ-treated primary rat-derived chondrocytes showed an increase of IκBα with a simultaneous decrease of phosphorylated IκBα in the cytosol at 40 µM 4,5-DCQA (Jang et al., 2022). Furthermore, nucleus translocation of p65 was evaluated in human- and rodent-based cell systems on protein level. Jang et al. (2022) observed simultaneously an increase of cytosolic p65 and decrease of p65 in the nucleus in stimulated RAW264.7 cells and primary rat-derived chondrocytes after treatment with 4,5-DCQA at concentrations of 4 and 20 µM (Jang et al., 2021). A decreased translocation of p65 in the nucleus was also observed within LPS-stimulated EA.hy926 cells after treatment with 5 µM 1,3-DCQA (Kim D. B. et al., 2022). Lastly, a decrease in phosphorylated p65, which is associated with enhanced transcription activity, in stimulated rodent-derived cell models after treatment with 3,5- and 4,5-DCQAs at concentrations of 100 and 10 μM, respectively, was observed (Zheng et al., 2022; Tang et al., 2023). Overall 4,5-DCQA seems to obtain a higher activity in regard of NF-κB deactivation, however data is limited to a few studies applying 3,5-DCQA to investigate this endpoint. Hence, no appropriate comparison regarding this endpoint can be made. Besides *in vitro* data, on respiratory tract specific study is available evaluating the potential of 3,5-DCQA to inhibit NF-κB activation *in vivo*. After intraperitoneal injection of 5 mg 3,5-DCQA to an acute lung injury model induced by LPS in mice resulted in a decrease of phosphorylated p65 within lung tissue, suggesting less NF-κB-dependent transcriptional activation (Wang and Xiao, 2019). All in all, these data indicates that DCQAs alleviates the activation of the NF-κB pathway on several levels potentially regardless of the isoform.

##### 4.2.3.2 Impact on MAPK pathway

The MAPK pathway represents a signaling pathway, which is involved in several cellular processes, including inflammation. The p38 subgroup of mitogen-activated protein kinases (MAPKs), which include also extracellular signal-regulated kinases (ERK) and c-Jun NH_2_-terminal kinases (JNK), are a group of MAPKs that are strongly activated by cytokines and chemokines (Figure 7). Further, activation of p38 MAPKs is suggested to be associated with the pathogenesis of COPD (Renda et al., 2008). Several studies investigated the potential of DCQA to alleviate the activation of the MAPK pathway after an induced inflammation by LPS *in vitro*. The level of phosphorylated p38, and therefore activated p38, was reduced in LPS-triggered RAW264.7 and EA.hy926 cells after treatment with 1,3- and 4,5-DCQA at concentrations of 1 and 4 μM, respectively (Jang et al., 2021; Kim D. B. et al., 2022). Furthermore, the levels of phosphorylated JNK and ERK in LPS-treated RAW264.7 cells were decreased by 3,5- and 4,5-DCQA at 1 and 4 μM, respectively (Jang et al., 2021; Yang et al., 2023). Taken together, these data indicates the potential of DCQAs to alleviate the activation of the MAPK signaling pathway, regardless of the applied isoform.

##### 4.2.3.3 Modulation of NLRP3 inflammasome

The NLRP3 (NLR family pyrin domain containing 3) inflammasome is a protein complex that belongs to the group of pattern recognition receptors, which recognize pathogen-associated molecular patterns (PAMPs) as well as damage-associated molecular patterns (DAMPs). These patterns subsequently activate mechanisms to eliminate the respective stimuli, e.g., infections, and/or repair tissue. Activation of the NLRP3 inflammasome occurs as response to various stimuli, e.g., pathogens. Briefly, the activation process itself is a two-step procedure. First, priming occurs via a variety of ligands resulting in an activation of NF-κB with subsequent enhanced NLRP3, pro-IL-1β, and pro-IL18 expression. Secondly, the protein complex of the inflammasome assembles, including the final activation of NLRP3 with subsequent cleavage of pro-IL-1β and pro-IL18 by caspase 1 to the respective active cytokines and their extracellular secretion. During the recent years an association between activation of NLRP3 inflammasome and inflammatory respiratory diseases has been made, including allergic rhinitis, asthma, and COPD (Leszczyńska et al., 2022). The potential of 1,3- and 3,5-DCQA to alleviate the activation of the NLRP3 inflammasome has been evaluated in two *in vivo* studies. Wang and colleagues (2022) applied 3,5-DCQA via intraperitoneal injection to an acute lung injury model in mice and observed a decrease of NLRP3 protein in lung tissue after injection of 5 mg 3,5-DCQA (Wang and Xiao, 2019). Furthermore, 25 mg/kg bw intraperitoneal injected 1,3-DCQA to an arthritis model in mice induced by monosodium urate resulted in decreased levels of NLRP3 protein in the respective paw tissue (Wu et al., 2022). Taken together, it can be suggested that DCQAs reduce the activation of NLRP3 inflammasome, however data is scarce and further research needs to be done to elucidate the impact of DCQAs on this inflammatory relevant pathway.

Inflammatory-associated pathways such as NLRP3, NF-κB, and MAPK play a crucial role in inflammation-related respiratory diseases such as acute/chronic bronchitis, asthma, or COPD (Mishra et al., 2018; Leszczyńska et al., 2022; Panek et al., 2023). Therefore, a treatment with agents which can decrease the activation of these pathways, such as DCQA, can have a positive impact in the treatment of such respiratory diseases.

### 4.3 Effects associated to respiratory diseases

This chapter will include investigated mechanisms or endpoints that are relevant for the treatment of respiratory diseases but are not categorized as antioxidative or anti-inflammatory effects.

Two *in vitro* studies investigated the inhibitory activity of DCQAs on the TRPV3 channel (transient receptor potential cation channel, subfamily V, member 3). Sun et al. (2020) observed inhibition rates of 81.8% and 90.6% by 50 µM 3,5- and 4,5-DCQA in HEK293 cells. Furthermore, IC_50_ values of 2.7 and 0.9 µM for 3,5- and 4,5-DCQA were observed in HEK293 cells (Qi et al., 2022). TRPV3, together with TRPV1, 2, and 4, is not only expressed in kidney cells, e.g., HEK293, but also in the laryngeal epithelium and is suggested to be involved in the genesis of cough (Hamamoto et al., 2008). The link between the inhibitory effect of DCQAs on TRPV3 can be further extrapolated to a reduction in coughs as Wu and colleagues (2016) observed less cough events after treatment with DCQAs in an ammonia liquor-induced cough model. Within this study, mice were treated with 10 and 20 mg/kg bw 3,4-, 3,5-, and 4,5-DCQA via oral administration once daily for 3 subsequent days followed by an inhalatory exposure to ammonium hydroxide and recording of the latent period and frequency of coughs for 2 min. As a result, all applied DCQAs increased the latent period of coughs already at 10 mg/kg bw up to 152%–195% (4,5-DCQA > 3,5-DCQA > 3,4-DCQA). Furthermore, cough events were significantly reduced compared to untreated mice at both, 10 and 20 mg/kg bw, and comparable with the positive control pentoxyverine (Wu et al., 2015). Comparing the different applied isoforms, no significant difference was observed. Furthermore, the study included a determination of the expectorant effects of the applied DCQAs via phenol red secretion in the described model. All applied DCQAs increased the secretion of phenol red compared to an untreated control already at the lower dose of 10 mg/kg bw; hence all DCQAs resulted in enhanced expectorant activities. Hereby, 3,5-DCQA increased phenol red secretion significantly more than the other DCQAs (Wu et al., 2015). The antitussive property and enhanced expectorant activity were also observed applying plant-based DCQAs-containing extracts orally to mice-based ammonia-induced cough models (Li et al., 2012; Li Z.-Y. et al., 2013; Huang et al., 2020). On a mechanistic level, phosphodiesterase (PDE) inhibitors are known to obtain beneficial properties for the treatment of respiratory diseases as they act as bronchodilators and anti-inflammatory (Satori et al., 2023; Stolfa and Page, 2023). An inhibition of cAMP-specific PDE activity in LXFL529L cells by 3,4-DCQA was observed beginning at approx. 100 μM resulting in an IC_50_ value roughly at 1 mM (Röhrig et al., 2017). This indicates that DCQAs obtain the potential to act as bronchodilators, which is further supported by studies identifying 3,5- and 4,5-DCQA as substances with antispasmodic properties applying the inhibition of explanted guinea-pig ileum contraction as endpoint (Trute et al., 1997; Capasso et al., 1998).

## 5 Discussion

### 5.1 Benefit of DCQA treatment during acute bronchitis

Acute bronchitis represents a self-limiting inflammatory disease of the tracheobronchial tract that is characterized by a dry or productive cough for less than 3 weeks duration. It is mostly of viral origin and one of the most frequent causes of medical consultations (Walsh, 2015). Furthermore, it is associated with a high symptom burden, hence respective therapeutics, including mucoactive agents, antihistamines, antitussives, and decongestants, mainly focus on relieving symptoms (Kardos, 2015). The pathogenesis of acute bronchitis includes, among other findings, submucosal congestion and mononuclear cell infiltration. Furthermore, occlusion of the airways and inflammatory changes in the respiratory tract were observed. From an immunological point of view, release of pro-inflammatory cytokines and chemokines are suggested to contribute to symptoms of systemic and local nature. In detail, INF-α, INF-γ, TNFα, IL-10 and IL-6 were enhanced in plasma after influenza virus infection (Kaiser et al., 2001). Furthermore, a correlation between increased plasma IL-6 as well as nasopharyngeal lavage levels of IL-6 and TNFα and symptom scores as well as temperature values was found (Kaiser et al., 2001). Due to this observation of a correlation between symptoms and the kinetics of cytokine secretion, an according therapy with anti-inflammatory acting substances seems beneficial. As summarized in chapter 4.2.1, *in vitro* and *in vivo* data indicated that various cytokines and inflammatory mediators were inhibited by DCQAs including IL-6 and TNFα, which seem to play a role during acute bronchitis. Therefore, the treatment of acute bronchitis with DCQAs containing extracts or DCQAs as single substance might help to reduce inflammatory processes during acute bronchitis and thus might be beneficial to relieve inflammation-associated symptoms. Further symptoms of acute bronchitis include dry or productive cough and at least to some degree airway obstruction. Accordingly, cough suppressants, expectorants, and bronchodilators in form of β_2_-agonsists are widely used (Walsh, 2015). As discussed in chapter 4.2.3, preclinical data on DCQAs suggested the ability to reduce cough events, enhance expectorant activity, and antispasmodic properties. In conclusion, DCQAs meet the needs to relieve symptoms associated with acute bronchitis and can be beneficial, as single substance or within plant-based extracts, as respective treatment.

### 5.2 Impact of DCQAs on chronic bronchitis

In contrast to acute bronchitis, the most used definition of chronic bronchitis describes this respiratory disease as chronic disease with sputum production for at least 3 months per year for two subsequent years (Agustí et al., 2023). The origin of chronic bronchitis are divers with smoking as the main risk factor followed by inhaled irritants such as dusts or fumes (Mejza et al., 2017). Even though viral infections are usually the cause of acute bronchitis, repeated infections can also provoke chronic bronchitis. Mucus overproduction resulting from inflammatory signals–also known as mucous metaplasia–is the pathological basis for chronic bronchitis. In combination with an impairment of the mucociliary clearance, e.g., reduction of bronchial cilia, this results in an airflow impairment due to obstruction of small airways. However, obstructed airways increase irritation resulting in enhanced inflammation leading again to mucous overproduction. Mucous metaplasia can be triggered by inflammatory mechanisms and has been extensively studied in the context of Th2-dependent cytokines IL-4, IL-5, and IL-13, which play an important role within asthma. A more relevant role for chronic bronchitis seems to be the secretion of IL-17A from Th17 cells. IL-17A induces the secretion of IL-6, which subsequently activates the transcription of two important airway mucins: MUC5AC and MUC5B (Kim and Criner, 2013). Furthermore, *MUC2*, *MUC5AC,* and *MUC5B* are upregulated by activated pathways such as NF-κB as well as MAPK and cytokines such as IL-1β or TNF-α (Thai et al., 2008). Similar to acute bronchitis, treatment options for chronic bronchitis focus on symptom relief during acute exacerbations in addition to deceleration of disease progression. Important aspects of therapy are the alleviation of mucus overproduction, controlling inflammation, increasing mucociliary clearance, and reducing cough. Accordingly, the use of short- and long-acting β-adrenergic receptor agonists and anticholinergics as bronchodilators and to improve mucociliary clearance, glucocorticoids to reduce inflammation and mucus production, and PDE-4 inhibitors to decrease inflammation and due to their bronchodilator properties represent the common treatment strategies for chronic bronchitis (Kim and Criner, 2013). As stated before, based on preclinical data the possibility of DCQAs to reduce cough events, increase expectorant activity as well as the potential to act as bronchodilator due to antispasmodic properties is indicated and was shown in chapter 4.2.3. Furthermore, antioxidant properties were identified in chapter 4.1, which may be beneficial not only by reducing cellular oxidative stress, but also potentially by reducing disulfide bonds linking mucin polymers, resulting in reduced sputum viscosity (Kim and Criner, 2013). In addition, the anti-inflammatory properties of DCQAs were evaluated in chapter 4.2 and included not only the inhibition of relevant mediators, i.e., IL-17A, IL-6, TNFα, IL-1β, but also the inhibition of inflammation-associated pathways, i.e., NF-κB and MAPK. As explained above, NF-κB and MAPK also play a relevant role within mucin secretion and hence respective inhibition might be beneficial to alleviate mucus overproduction. In summary, based on the presented preclinical data DCQAs–as single substance or within plant-based extracts–indicate to have the potential to provide a benefit in the symptomatic therapy of chronic bronchitis.

### 5.3 Potential benefit of DCQAs in the treatment of COPD

The Global Initiative for Chronic Obstructive Lung Disease defines COPD in their 2023 report as a “*heterogeneous lung condition characterized by chronic respiratory symptoms (dyspnea, cough, expectoration and/or exacerbations) due to abnormalities of the airways (bronchitis, bronchiolitis) and/or alveoli (emphysema) that cause persistent, often progressive, airflow obstruction.*” (Agustí et al., 2023) This suggests that chronic bronchitis, and in particular its symptoms, can be a part of COPD. Therefore (environmental) risk factors, e.g., smoking, occupational inhalation of dusts and fumes, pathology, and pharmacological treatment are similar, except for the occurrence of emphysema within COPD (Agustí et al., 2023; Panek et al., 2023). Therefore, all arguments based on preclinical data for the benefits of treatment with DCQAs in plant-based extracts or as single substance for chronic bronchitis may also be valid for COPD. In addition, the NLRP3 inflammasome-dependent processes are suggested to play an important role in the development of COPD and potentially asthma (Leszczyńska et al., 2022; Panek et al., 2023). Briefly, the exposure to cigarette smoke to mice results in the development of COPD alike pathologies, whereas the same exposure in NLRP3-knock out mice did not develop pathophysiological characteristics of COPD (Panek et al., 2023). Furthermore, NLRP3 inflammasome seems to be involved in the development of acute exacerbations of COPD (Panek et al., 2023). Therefore, downregulation of NLRP3 inflammasome, as it was observed for DCQAs, might be beneficial within the symptomatic treatment of COPD patients.

### 5.4 Relationship between asthma and the pharmacological activity of DCQAs

Asthma is a chronic inflammatory and heterogeneous airway disease with airway obstruction and airway hyperresponsiveness as its hallmarks (Lambrecht et al., 2019; Habib et al., 2022). Latter results in episodic and reversible bronchoconstriction which can be caused by allergens. Furthermore, asthma results in tissue remodeling and excessive mucus production (Figueiredo et al., 2023). This disease has mainly been associated with Th2 cell cytokines, i.e., IL-4, IL-5, and IL-13, however recent developments have led to a terminology of Th2-high and Th2-low asthma (Lambrecht et al., 2019; Habib et al., 2022). Briefly, as the induction of asthma is a complex topic, Th2-high asthma represents the mechanism of IL-4, IL-5, and IL-13 associated asthma, which induces roughly 50% of mild-to-moderate asthma and the majority of severe asthma inflammation (Habib et al., 2022). In contrast, Th2-low asthma is mediated by IL-17 and associated with IL-6, which is related to Th17 differentiation and therefore IL-17 secretion, and TNFα (Ramakrishnan et al., 2019; Habib et al., 2022). So far, asthma treatment is based on two streams. On the one hand, the use of bronchodilators to directly inhibit bronchoconstriction and take control of asthma attack resulting in relieving respective symptoms. On the other hand, anti-inflammatory drugs, e.g., corticosteroids, are applied to decrease chronic inflammation (Figueiredo et al., 2023). While the authors do not suggest that DCQAs, alone or within a plant-based extract, are useful to relief rapidly occurring asthma attacks, a co-medication with other anti-inflammatory drugs might be beneficial due to DCQAs ability to decrease inflammatory mediators playing a role within asthma, such as IL-17, IL-6, or TNFα.

### 5.5 Evaluation of a possible isomer-specific pharmacological activity

This review does not suggest a significant biological difference in the pharmacological activities of the different DCQA isoforms. Although it is known that monocaffeoylquinic isomers exhibit varying activities in sensitization following intravenous injection (Lin et al., 2012), this review specifically focuses on oral intake. Zhao et al. (2022) have demonstrated that DCQAs undergo extensive metabolism into up to 67 metabolites when administered orally to rats. Their research indicates that DCQAs are hydrolyzed into quinic and caffeic acids, which then undergo processes such as methylation, hydrogenation, hydration, dehydroxylation, sulfatation, and glucuronation. This metabolic pathway is hypothesized to be at least comparable across the different DCQA isoforms (Zhao et al., 2022). Consequently, the authors suggest no significant difference in pharmacological activity between DCQA isoforms after oral intake even if a metabolite is the cause for the pharmacological activity. However, as there are no clinical studies available to substantiate this suggestion, further research is required to confirm this hypothesis.

## 6 Conclusion

Dicaffeoylquinic acids are constituents of various medicinal plants, such as *Hedera helix*, which are used to treat inflammatory diseases of the respiratory tract. Within the multi-constituent composition of plant-derived extracts, it is suggested that DCQAs play an important role in their respective pharmacological activities. This review examined the antioxidant potential of DCQAs both *in vitro* and *in vivo* with respect to their ability to scavenge free radicals and enhance cellular oxidative defense. Furthermore, it was shown that DCQAs are able to downregulate several important cytokines and inflammatory mediators relevant in acute and even chronic respiratory diseases. The anti-inflammatory activity was further strengthened by the ability to deactivate crucial pathways associated with inflammation, such as NF-κB. Finally, specific endpoints relevant to respiratory diseases were evaluated and revealed that DCQAs reduced symptoms of inflammation-associated respiratory diseases. Specifically, enhanced expectorant activity, reduction of cough, and antispasmodic properties were observed. Taken together, the data implies that DCQAs as constituents of medicinal plant extracts possibly contribute to their proven efficacy in the treatment of respiratory diseases. Further preclinical studies on the mechanism of action, possible influences on other signaling pathways, and identification of the actual pharmacological active substance–DCQA or metabolite–are necessary to fully understand the pharmacological profile and the therapeutic potential of DCQAs. In particular, more clinical studies of DCQA-containing extracts should be conducted to prove the importance of such phytopharmaceuticals in the treatment of respiratory diseases.

## Figures and Tables

**Table:** 

**ABTS**	2,2′-azino-bis(3-ethylbenzothiazoline-6-sulfonic acid)
**AUC**_**0-∞**_	area under the concentration-time curve from zero to infinity
**BALF**	bronchoalveolar lavage fluid
**BMDMs**	bone marrow-derived macrophages
**CAT**	Catalase
**c**_**max**_	maximum concentration
**COPD**	chronic obstructive pulmonary disease
**DCF**	Dichlorofluorescin
**DCQA**	dicaffeoylquinic acid
**DPPH**	2,2-diphenyl-1-picrylhydrazyl
**ERK**	extracellular singal-regulated kinases
**f**	Bioavailability
**FRAP**	ferric ion reducing antioxidant power
**G6PD**	glucose-6-phosphate dehydrogenase
**GPX**	glutathione peroxidase
**GR**	glutathione reductase
**GSH**	Glutathione
**HO-1**	heme oxygenase
**iNOS**	inducible nitric oxide synthase
**JNK**	c-Jun NH_2_-terminal kinases
**LPS**	Lipopolysaccharide
**MAPK**	mitogen-activated protein kinase
**MDA**	Malondialdehyde
**NASH**	non-alcoholic steatohepatitis
**NLRP3**	NLR family pyrin domain containing 3
**NOS**	nitric oxide synthase
**PDE**	Phosphodiesterase
**PGE**_**2**_	prostaglandin E_2_
**PoD**	point of departure
**SOD**	superperoxide dismutase
**TBHP**	tert-butylhydroperoxide
**TRPV3**	transient receptor potential cation channel, subfamily V, member 3
**t**_**max**_	time to reach maximum concentration
